# Transcriptome response comparison between vector and non-vector aphids after feeding on virus-infected wheat plants

**DOI:** 10.1186/s12864-020-07057-0

**Published:** 2020-09-15

**Authors:** Dandan Li, Chi Zhang, Zeqian Tong, Dan Su, Gaisheng Zhang, Shize Zhang, Huiyan Zhao, Zuqing Hu

**Affiliations:** 1 State Key Laboratory of Crop Stress Biology in Arid Areas, College of Plant Protection, Northwest A&F University, Yangling, China; 2 Ministry of Education, Key Laboratory of Crop Heterosis of Shaanxi Province, National Yangling Agricultural Biotechnology and Breeding Center, Yangling Branch of State Wheat Improvement Centre/Wheat Breeding Engineering Research Center, Northwest A&F University, Yangling, China

**Keywords:** BYDV, WDV, Vector, Non-vector, Transcriptome response, Gene clone

## Abstract

**Background:**

Plant viruses maintain intricate interactions with their vector and non-vector insects and can impact the fitness of insects. However, the details of their molecular and cellular mechanisms have not been studied well. We compared the transcriptome-level responses in vector and non-vector aphids (*Schizaphis graminum* and *Rhopalosiphum padi*, respectively) after feeding on wheat plants with viral infections (*Barley Yellow Dwarf Virus* (BYDV) and *Wheat dwarf virus* (WDV), respectively). We conducted differentially expressed gene (DEG) annotation analyses and observed DEGs related to immune pathway, growth, development, and reproduction. And we conducted cloning and bioinformatic analyses of the key DEG involved in immune.

**Results:**

For all differentially expressed gene analyses, the numbers of DEGs related to immune, growth, development, reproduction and cuticle were higher in vector aphids than in non-vector aphids. STAT5B (signal transducer and activator of transcription 5B), which is involved in the JAK-STAT pathway, was upregulated in *R. padi* exposed to WDV. The cloning and bioinformatic results indicated that the *RpSTAT5B* sequence contains a 2082 bp ORF encoding 693 amino acids. The protein molecular weight is 79.1 kD and pI is 8.13. Analysis indicated that *RpSTAT5B* is a non-transmembrane protein and a non-secreted protein. Homology and evolutionary analysis indicated that *RpSTAT5B* was closely related to *R. maidis.*

**Conclusions:**

Unigene expression analysis showed that the total number of differentially expressed genes (DEGs) in the vector aphids was higher than that in the non-vector aphids. Functional enrichment analysis showed that the DEGs related to immunity, growth and reproduction in vector aphids were higher than those in non-vector aphids, and the differentially expressed genes related to immune were up-regulated. This study provides a basis for the evaluation of the response mechanisms of vector/non-vector insects to plant viruses.

## Background

*Schizaphis graminum* and *Rhopalosiphum padi* are serious pests of cereal crops, particularly wheat that may cause harm to plants by feeding on them and by transmitting the *Barley yellow dwarf virus* (BYDV) (Luteoviridae: Luteovirus) in a persistent and non-proliferative manner [1–4]. That causes one of the most economically important viral diseases of cereal plants [5, 6]. In China, there were 4 isolated strains be identified as GPV, GAV, PAV and RMV and the BYDV-GAV is the major isolate [7]. *Wheat dwarf virus* (WDV) (Geminiviridae: Mastrevirus) is another serious virus of wheat in China that causes significant losses and is mainly transmitted by the leafhopper *Psammotettix alienus* (Dahlbom) in a persistent and non-proliferative manner [8, 9]. *S. graminum* is a vector of BYDV-GAV and is a non-vector of WDV, while *R. padi* is a non-vector of both BYDV-GAV and WDV.

The aphids (*S. graminum* and *R. padi*) and viruses (BYDV-GAV and WDV) used in our study are important insect pests and viral pathogens that often occur together on the same host plant in agro-ecosystems. The plant-arthropod-pathogen interactions are complex, and some studies have shown that plant viruses can produce favorable or unfavorable effects on vector/or non-vector insects [10, 11]. Exploring the effect of viruses on aphids can provide a better understanding of these three-way interactions and improve integrated pest management.

Previous studies showed that plant virus infection of its host plant could result in either beneficial or adverse effects on its vector via direct feeding or other indirect ways [12, 13]. Transcriptional induction or repression is an important mechanism for insects to regulate their innate immune responses [14]. There are a few previous reports that have studied the global transcription profiles of insect vectors fed on virus-infected plants. Brault [15] conducted the first large-scale analysis of aphid gene regulation following virus acquisition. *Southern rice black-streaked dwarf virus* (SRBSDV) infection activated the immune regulatory systems of the white-backed planthopper (WBPH) [16]. Transcriptome analysis of the thrips response to *Tomato spotted wilt virus* (TSWV) infection showed that the diversity of the innate immune-related transcripts in response to viral infection was most pronounced in the P1 stage [17]. Reproduction, embryo development and growth were associated with upregulated contigs in virus-exposed *Frankliniella fusca* adults [18].

Compared with vectors, there are fewer studies on non-vectors. Although less studied, there is some evidence that plant viruses have an impact on non-vectors. The nymph survival rate was decreased and the longevity of female adults was shortened, while egg hatchability increased in response to non-vector brown planthoppers feeding on SRBSDV-infected plants [19]. In contrast, rice plants infected by *R* *ice black-streaked dwarf virus* (RBSDV) improved the ecological fitness of the brown planthopper [20]. In addition, TSWV infection reduced the fecundity and longevity of *B. tabaci* and increased the developmental time of *Bemisia tabaci* [21]. Additionally, feeding on plants inoculated with TSWV enhanced the developmental and oviposition rates of spider mites [22].

Pathogenic infections may affect the physiology of the host insect and even cause death [23]. In the process of long-term evolution, insects have formed defense mechanisms to achieve a dynamic balance between viruses and insects. Insects rely on their immune system to resist the invasion of pathogens, which is not conducive to the replication and transmission of the virus. One of the long-term goals of studying virus-insect interactions is to elucidate the molecular mechanism by which viruses affect the innate immune system of insects to avoid damage from the immune defense response. Therefore, understanding the insect’s immune system is key to solving this problem. Immune-related signaling pathways that have been studied in insects include the RNAi, Toll, JAK/STAT, phagocytosis, apoptosis, proteolysis and JNK pathways [18, 24, 25].

Because the persistent plant viruses cannot break through the midgut barrier or salivary gland barrier of non-vector insects, they cannot be transmitted by the non-vector insects [26]. We previously have found that plant virus (e.g., BYDV-GAV and WDV) can be detected in non-vector aphids (e.g., *R. padi*) (unpublished data). Previous studies aslo have shown that plant viruses can improve the fitness of non-vector insects (as well as vectors) [20–23]. We speculate that plant viruses in non-vector aphids can also affect their biochemical response. At present, the molecular and cytological mechanism of plant viruses to the vector and non-vector insects is not clear, so BYDV-GAV and WDV were used as the test viruses, *R. padi* and *S. graminum* were used as the test insects to compare the transcriptome changes of the vector and non-vector insects to the plant viruses, so as to find out the differences between the plant viruses affecting the gene expression of the vector and non-vector insects. This study will lay the foundation for the molecular mechanism of plant viruses to improve the fitness of non-mediator insects.

## Results

### Sequence assembly and annotation

To explore the transcriptome response to viral infection by vector and non-vector aphids*,* we performed RNA-Seq analysis on adult *S. graminum* and *R. padi* exposed to BYDV-infected, WDV-infected and uninfected wheat plants. The *S. graminum* and *R. padi* cDNA libraries were sequenced, which generated 72,092 and 68,996 unigenes, respectively (Table 1). The N50 sizes were 1481 kb and 1497 kb, respectively. The Q30 of each transcriptome library was above 85.74 and 85.62% for *S. graminum* and *R. padi*, respectively.

**Table 1:** Summary statistics for *S. graminum* and *R. padi* transcriptome assembly

Statistics	*S. graminum*	*R. padi*
Total number of transcripts	160,522	142,240
Total number of unigenes	72,092	68,996
Unigenes length > 1 kb	13,758	12,588
Mean length of unigenes	833.11	805.47
N50 unigene length	1481	1497

**Fig. 1:**
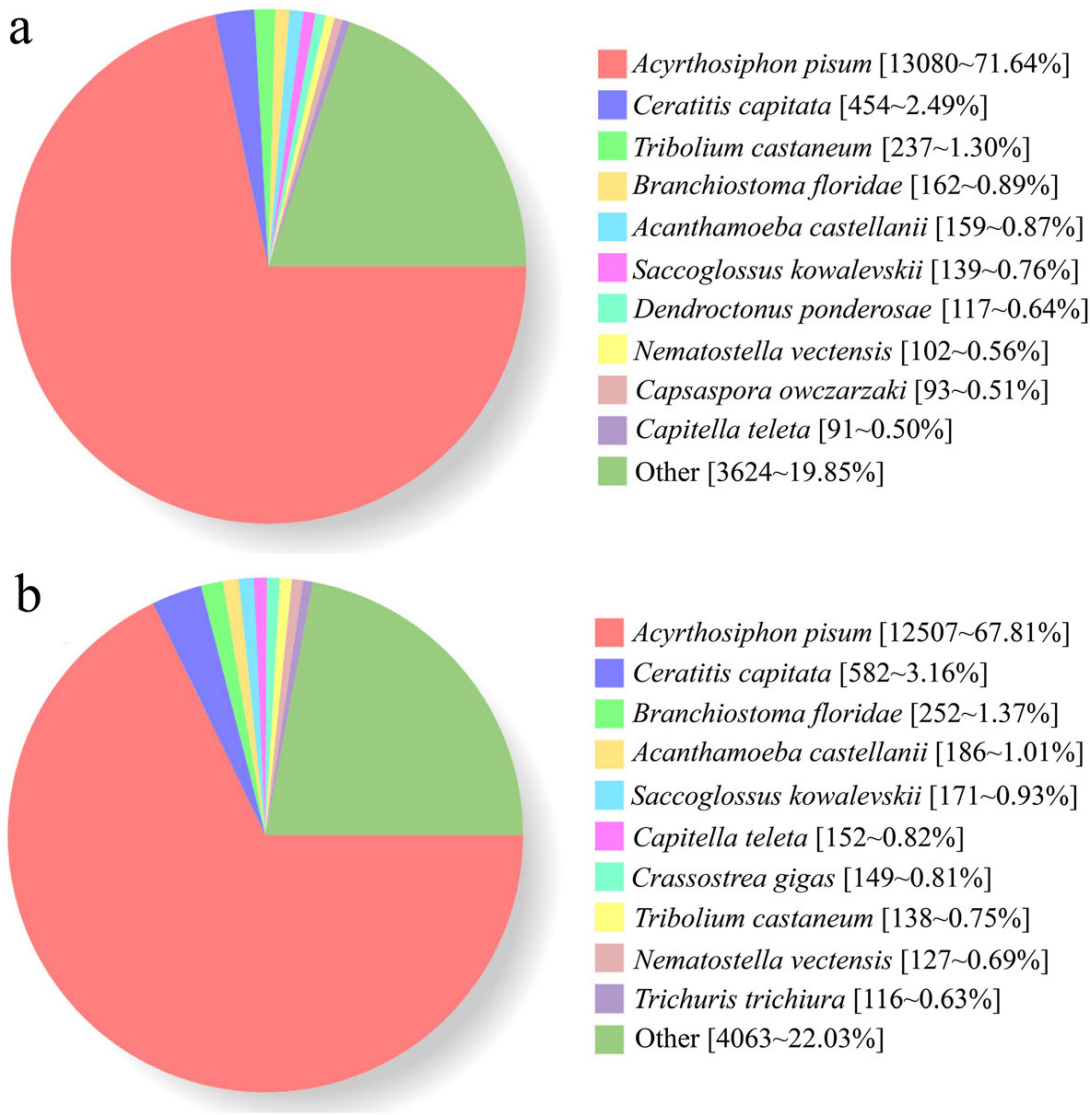
Nr homologous species distribution of *S. graminum* **a** and *R. padi* **b**

Nr (non-redundant protein sequence database) homologous species distribution revealed that 71.64% of *S. graminum* sequences matched with *Acyrthosiphon pisum* (Fig. 1 a), and 67.81% of *R. padi* sequences matched with *A. pisum* (Fig. 1 b). For the Gene ontology assignment, 7263 and 8164 unigenes in *S. graminum* and *R. padi* could be annotated. For *S. graminum,* 6200 unigenes were associated with biological process, 3080 with cellular component, and 6344 with molecular function (Fig. S1 A). In *R. padi,* 7104 unigenes were associated with biological process, 3597 with cellular component, and 7173 with molecular function (Fig. S1 B). These sequences have been submitted to the NCBI Sequence Read Archive (SRA), and the accession number is PRJNA490258.

### DEGs (Differentially expressed genes) in *S. graminum* and *R. padi*, as the vector/non-vector, in response to feeding on virus-infected wheat plants

**Fig. 2:**
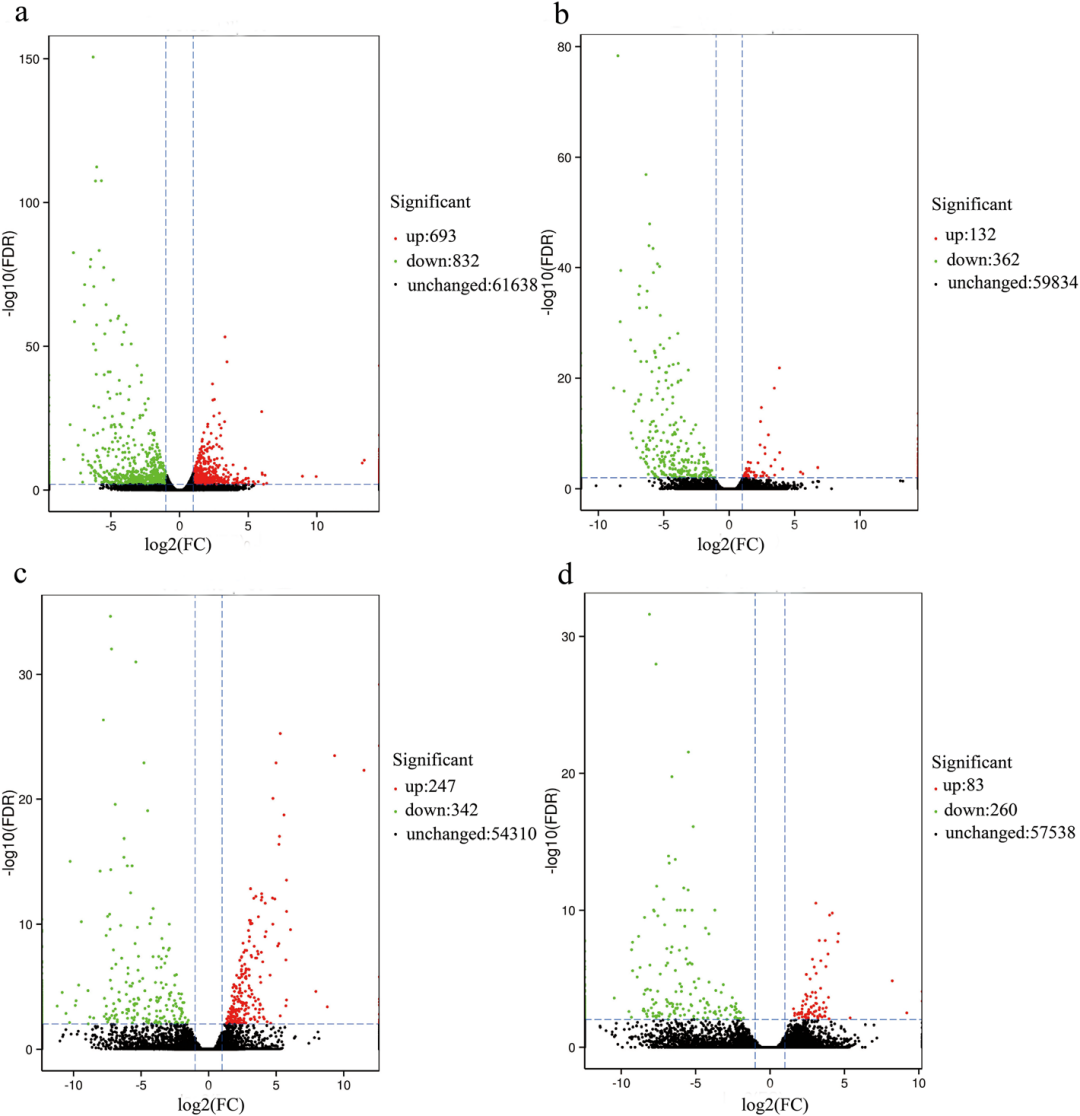
Volcano Plot of DEGs in *S. graminum* fed on wheat infected with BYDV **a**, *S. graminum* fed on wheat infected with WDV **b**, *R. padi* fed on wheat infected with BYDV **c** and *S. graminum* fed on wheat infected with BYDV **d**

For *S. graminum* fed on BYDV-infected wheat, 1525 DEGs were identified, with 693 upregulated and 832 downregulated. There were fewer DEGs in the *S. graminum* fed on WDV-infected wheat (494), and the majority were downregulated (73.28%). There were 589 DEGs in *R. padi* exposed to BYDV-infected wheat, with 247 upregulated and 342 downregulated. A total of 343 DEGs were identified in *R. padi* exposed to WDV-infected wheat, with 75.8% downregulated. The change ratios of most DEGs were between 2^− 5^ and 2^5^ (Fig. 2). Gene ontology (GO) and Kyoto Encyclopedia of Genes and Genomes (KEGG) enrichment analyses were conducted for all DEGs.

### Distribution and enrichment of DEGs in vector and non-vector aphids into gene ontologies (GOs)

**Fig. 3:**
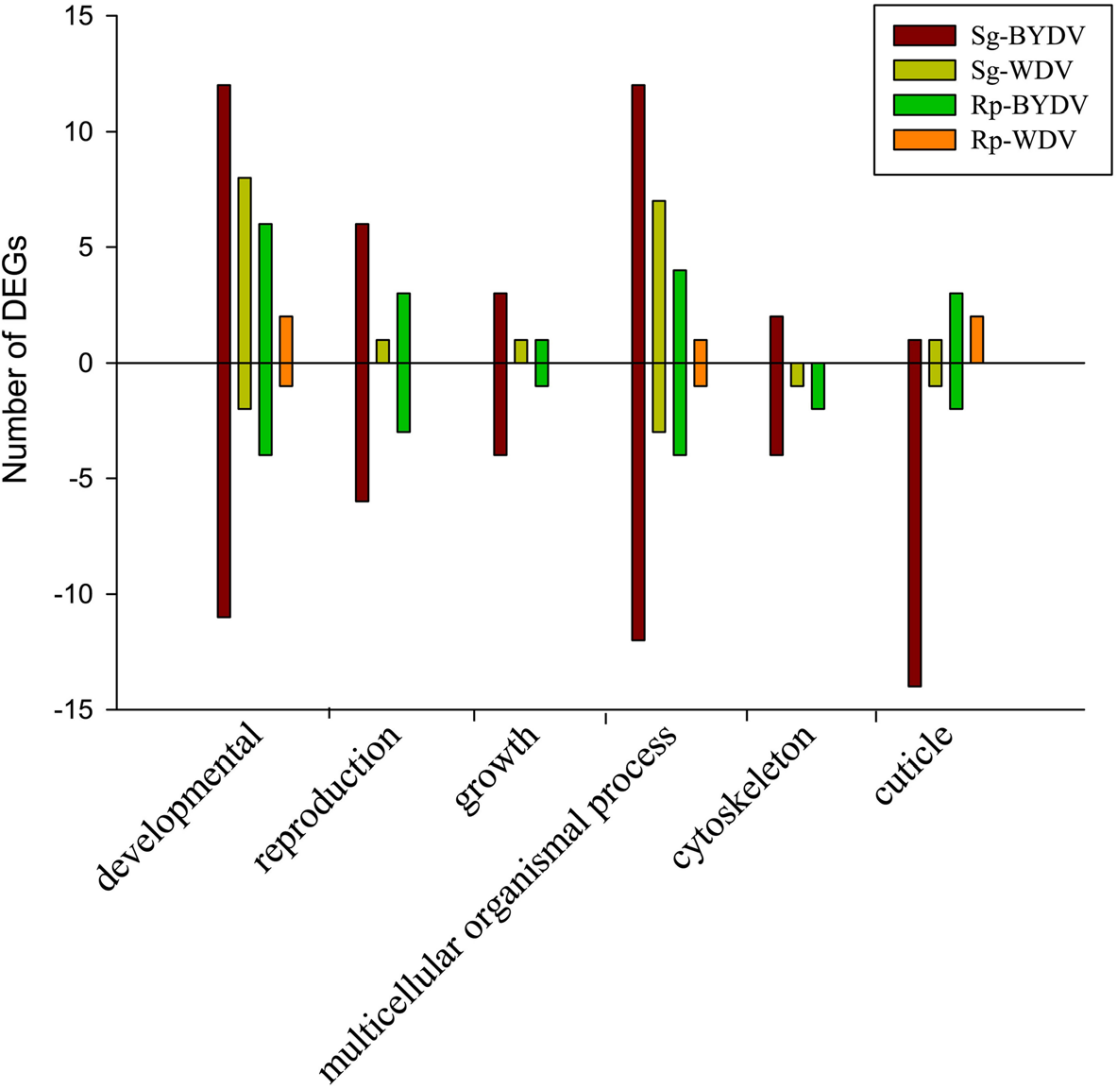
Number of differentially expressed genes (DEGs) involved in the development, reproduction, growth, multicellular organismal process, cytoskeleton and cuticle of vector (Sg-BYDV) and non-vector (Sg-WDV, Rp-BYDV, and Rp-WDV) aphids

Gene ontology (GO) analysis of all the DEGs in *S. graminum* and *R. padi* was conducted to classify the sequences into the biological process, molecular function and cellular component categories (Figs. S2, S3, S4, S5). The most enriched category in both vector and non-vector aphids was metabolic process (Figs. S2, S3, S4, S5). Among all comparisons, the differentially expressed genes contained a high proportion of genes involved in development, metabolism, reproduction and growth, and the number in vector aphids was higher (29 DEGs in Sg-BYDV) than that in non-vector aphids (13, 13, and 3 DEGs in Sg-WDV, Rp-BYDV, and Rp-WDV, respectively) (Fig. 3 and Table S5). In addition, genes associated with the cuticle (structural constituent of the cuticle and structural constituent of the chitin-based cuticle) were also differentially expressed, and there were 16 DEGs in the vector treatment (Sg-BYDV), while there were fewer in the non-vector treatments (with 2 in Sg-WDV, 5 in Rp-BYDV, and 2 in Rp-WDV). In Sg-BYDV, there were 15 DEGs associated with the cuticle, and the majority of them were downregulated (14 downregulated) (Fig. 3 and Table 2). Several genes involved in the cytoskeleton were also differentially expressed, with 6, 1, and 2 related DEGs in Sg-BYDV, Sg-WDV, and Rp-BYDV, respectively (Fig. 3 and Table 3). The results of GO enrichment analysis showed that the number of categories of DEGs enriched in vector aphids was higher than that in non-vector aphids (Figs. S2, S3, S4, S5). The Clusters of Orthologous Groups of proteins (COG) enrichment analysis showed a similar pattern in that the number of categories in the vector aphids was greater than that in the non-vector aphids (Figs. S6, S7, S8, S9).

**Table 2:** DEGs involved in the cuticle in vector (Sg-BYDV) and non-vector (Sg-WDV, Rp-BYDV, Rp-WDV and Rp-WDV) aphids

	Gene ID	Annotation	log2FC	FDR
Sg-BYDV	Sg39301	structural constituent of cuticle	− 1.646	8.07E-05
	Sg27246	structural constituent of cuticle	−1.028	5.62E-07
	Sg19143	structural constituent of cuticle	−1.872	6.79E-08
	Sg56253	structural constituent of cuticle	−2.454	7.80E-14
	Sg17654	structural constituent of cuticle	2.147	1.02E-06
	Sg27360	structural constituent of cuticle	−1.646	1.08E-15
	Sg56455	structural constituent of cuticle	−1.722	0.001659
	Sg56015	structural constituent of cuticle	−1.178	0.000655
	Sg35648	structural constituent of cuticle	−1.328	1.59E-08
	Sg53861	structural constituent of cuticle	−1.740	0.00481
	Sg47817	structural constituent of cuticle	−2.061	0.002456
	Sg36013	structural constituent of cuticle	−1.347	0.001048
	Sg54274	structural constituent of cuticle	−1.190	7.25E-12
	Sg55542	structural constituent of cuticle	−1.864	0.000179
	Sg30508	structural constituent of chitin-based cuticle	−1.155	0.001031
Sg-WDV	Sg17654	structural constituent of cuticle	2.458	2.12E-15
	Sg54274	structural constituent of cuticle	−1.892	0.00097
Rp-BYDV	Rp23024	structural constituent of cuticle	2.045	0.000696
	Rp23495	structural constituent of cuticle	−2.608	0.000335
	Rp37328	structural constituent of cuticle	−2.086	9.10E-05
	Rp18153	structural constituent of cuticle	4.732	8.00E-13
	Rp22931	structural constituent of chitin-based cuticle	3.899	1.19E-12
Rp-WDV	Rp48839	structural constituent of cuticle	2.603	0.001872
	Rp18153	structural constituent of cuticle	3.776	0.001354

Annotation: determined by BLAST

**Table 3:** DEGs involved in cytoskeleton

	Gene ID	Annotation	log2FC	FDR
Sg-BYDV	Sg53134	Cytoskeleton	1.098	3.88E-08
	Sg55484	Cytoskeleton	−2.175	3.45E-05
	Sg32025	Cytoskeleton	Inf	8.62E-08
	Sg56027	Cytoskeleton	−1.346	0.000376
	Sg49676	Cytoskeleton	−1.527	0.008734
	Sg55690	cytoskeleton	−1.879	5.80E-18
Sg-WDV	Sg55690	Cytoskeleton	−2.033	7.56E-07
Rp-BYDV	Rp29892	Cytoskeleton	−4.719	2.19E-05
	Rp50802	Cytoskeleton	−3.857	0.000136

### KEGG pathways analysis

To further understand the metabolic pathways that contained differentially expressed genes, KEGG significant enrichment analysis was performed on the differentially expressed genes between virus-treated vector and non-vector aphids. Among all of the DEGs, there were 287, 74, 91 and 27 DEGs that were assigned to KEGG pathways in Sg-BYDV, Sg-WDV, Rp-BYDV and Rp-WDV, respectively (Fig. S10, S11, S12, S13). The DEGs of the richest and shared pathways were associated with metabolism, including translation and fatty acid and carboxylic acid metabolic pathways. Genes involved in energy production pathways may be important for immune defense responses [27]. In the KEGG pathway analyses, we also identified DEGs involved in immune pathways.

### Immune-related DEGs of vector and non-vector aphids feeding on virus-infected wheat

Feeding on virus-infected plants resulted in DEGs related to the innate immunity pathway being upregulated. The DEGs related to immunity included several members of the pathway of the lysosomes, the JAK-STAT signaling pathway, antigen processing and presentation, ubiquitin mediated proteolysis, and the peroxisome (Table 4). For the DEG analyses, we identified 12 DEGs involved in immune pathways in the vector aphids (Sg-BYDV), the majority of which were upregulated (11 DEGs). For the non-vector aphids, there were fewer DEGs related to immunity, and only one DEG involved in immunity was identified in each of the Sg-WDV, Rp-BYDV, and Rp-WDV conditions (Table 4).

**Table 4:** Immune-related DEGs expressed in virus-infected *S. graminum* and *R. padi*

Treatment	Gene ID	Gene Name	Pathways	log2FC	FDR
Sg-BYDV	Sg55104	PIAS1	JAK-STAT signaling pathway	1.305	0.00022237
	Sg50416	UBE2D	Ubiquitin mediated proteolysis	5.042	0.001199371
	Sg49574	BIRC2_3	Ubiquitin mediated proteolysis	1.164	4.42E-09
	Sg55104	PIAS1	Ubiquitin mediated proteolysis	1.304	0.00022237
	Sg42560	HAO	Peroxisome	1.287	6.39E-08
	Sg43235	cathepsin	Lysosome	3.728	4.94E-06
	Sg45052	cathepsin	Lysosome	4.559	0.00263105
	Sg47282	cathepsin	Lysosome	2.494	2.06E-05
	Sg56271	cathepsin	Lysosome	−2.725	1.22E-05
	Sg53565	Legumain	Lysosome	2.621	4.84E-08
	Sg40066	LAMAN	Lysosome	1.223	2.49E-07
	Sg55760	NPC2	Lysosome	1.436	0.009359831
Sg-WDV	Sg40066	LAMAN	Lysosome	1.312	2.49E-07
Rp-BYDV	Rp46740	LAMAN	Lysosome	1.514	0.00928585
Rp-WDV	Rp49092	STAT5B	JAK-STAT signaling pathway	2.138	0.00295669

FC (fold change): calculated by RPKM

*FDR* false discovery rate

### RT-qPCR

**Fig. 4:**
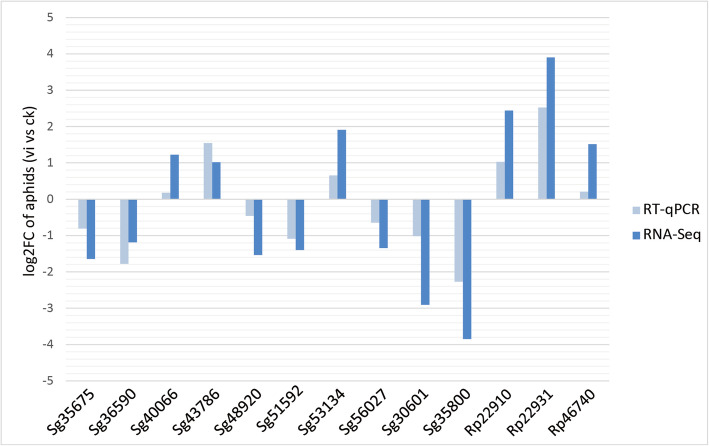
Quantitative reverse transcription-polymerase chain reaction (RT-qPCR) validation of selected genes differentially regulated in *S. graminum* and *R. padi* fed on virus-infected wheat (vi) compared with a virus-free control (ck) *S. avenae*

RT-qPCR was used to confirm the expression of 14 selected DEGs of *S. graminum* and *R. padi*. These genes were selected because we were interested in their function, since they were involved in immunity, development, growth and reproduction. Based on the transcriptome data, ten unigenes, namely, Sg35675 (Synaphin protein), Sg36590 (Immunoglobulin domain), Sg40066 (Glycosyl hydrolases family), Sg43786 (plexin A3-like), Sg48920 (Immunoglobulin domain), Sg51592 (transcriptional regulator CRZ2-like), Sg53134 (Cadherin domain), Sg56027 (Repeat in HS1/Cortactin), Sg30601 (AAA domain), Sg35800 (PDZ domain) were highly up- or down-regulated in virus-exposed *S. graminum*. The remaining six unigenes, Rp22910 (apolipophorin-3-like precursor), Rp22931 (cuticular protein 68 precursor), Rp46740 (lysosomal alpha-mannosidase-like), Rp49092 (STAT protein), were highly expressed in virus-exposed *R. padi*. The results of RT-qPCR showed that all 14 selected genes showed the same expression patterns (upregulated or downregulated) as the RNA-Seq analyses (Fig. 4).

### Cloning and characterization of STAT5B in *R. padi*

For the immunity related genes analysis, STAT5B (signal transducer and activator of transcription 5B) was the only related gene that was upregulated in *R. padi* fed on WDV infected wheat. STAT5B is a key gene involved in the JAK-STAT pathway, which is important for the innate immune response of insects. Based on the sequence of the transcriptome, we cloned STAT5B from *R. padi* (designated as *RpSTAT5B*)*.* The cloned product was validated using RT-PCR and sequencing. The sequencing results of the *RpSTAT5B* individual isolated colonies confirmed a single transcript identical to that found in the transcriptome sequencing. We have submitted the sequence of STAT5B to the NCBI GenBank database (accession number MK931299). The cloned product of *RpSTAT5B* is 2364 bp, and it has a complete open reading frame of 2082 bp encoding 693 amino acids, with a molecular weight of 83.2 kDa and a theoretical isoelectric point of 8.31.

**Fig. 5:**
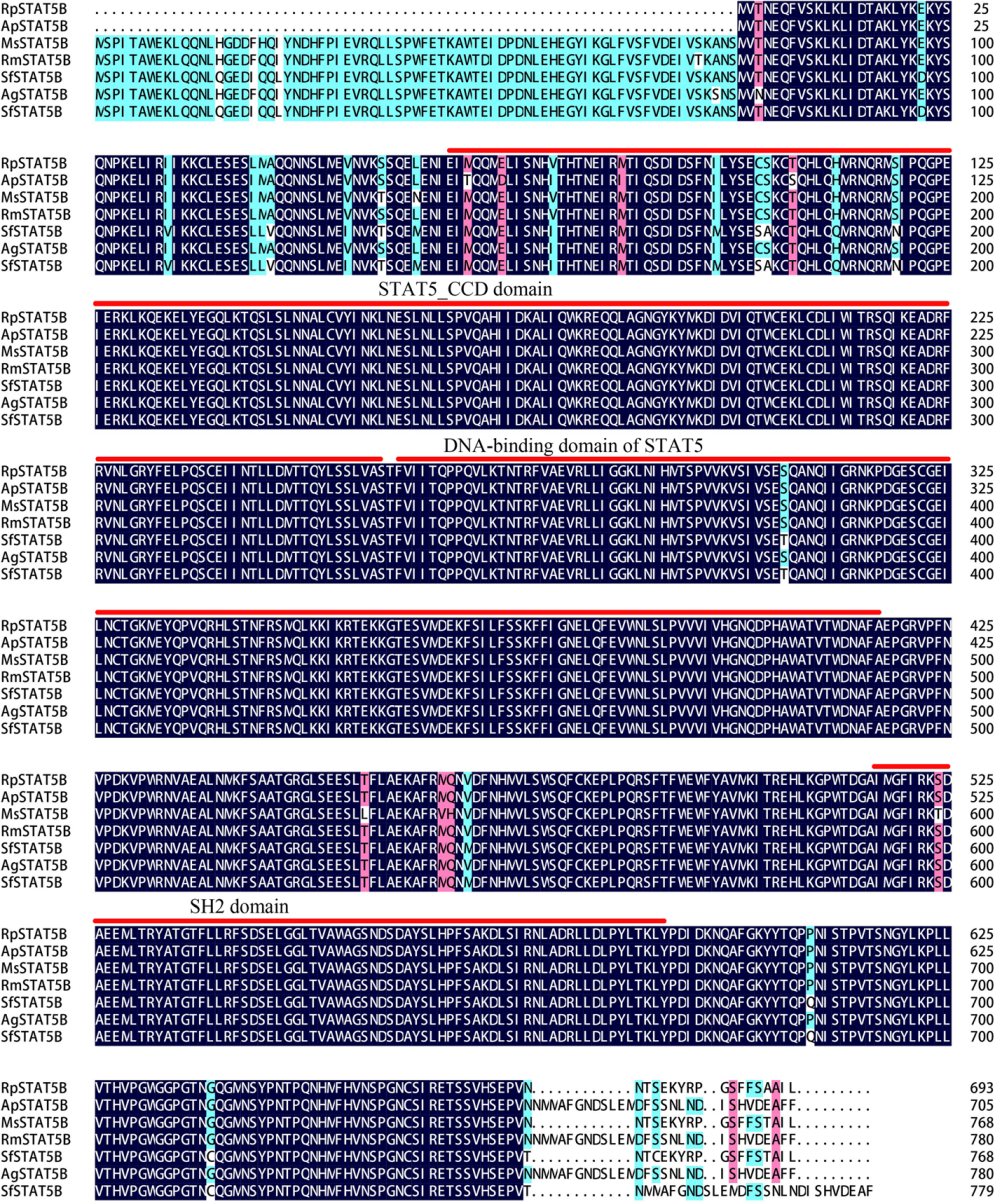
*RpSTAT5B* amino acid sequence alignment with other STAT5Bs from other insects. ApSTAT5B: *Acyrthosiphon pisum*, XP_008188159.1; MsSTAT5B: *Melanaphis sacchari,* XP_025209095.1; RmSTAT5B: *Rhopalosiphum maidis*, XP_026814403.1; SfSTAT5B: *Sipha flava*, XP_025407855.1; AgSTAT5B: *Aphis gossypii*, XP_027841453.1; SfSTAT5B: *S. flava*, XP_025407854.1

**Fig. 6:**
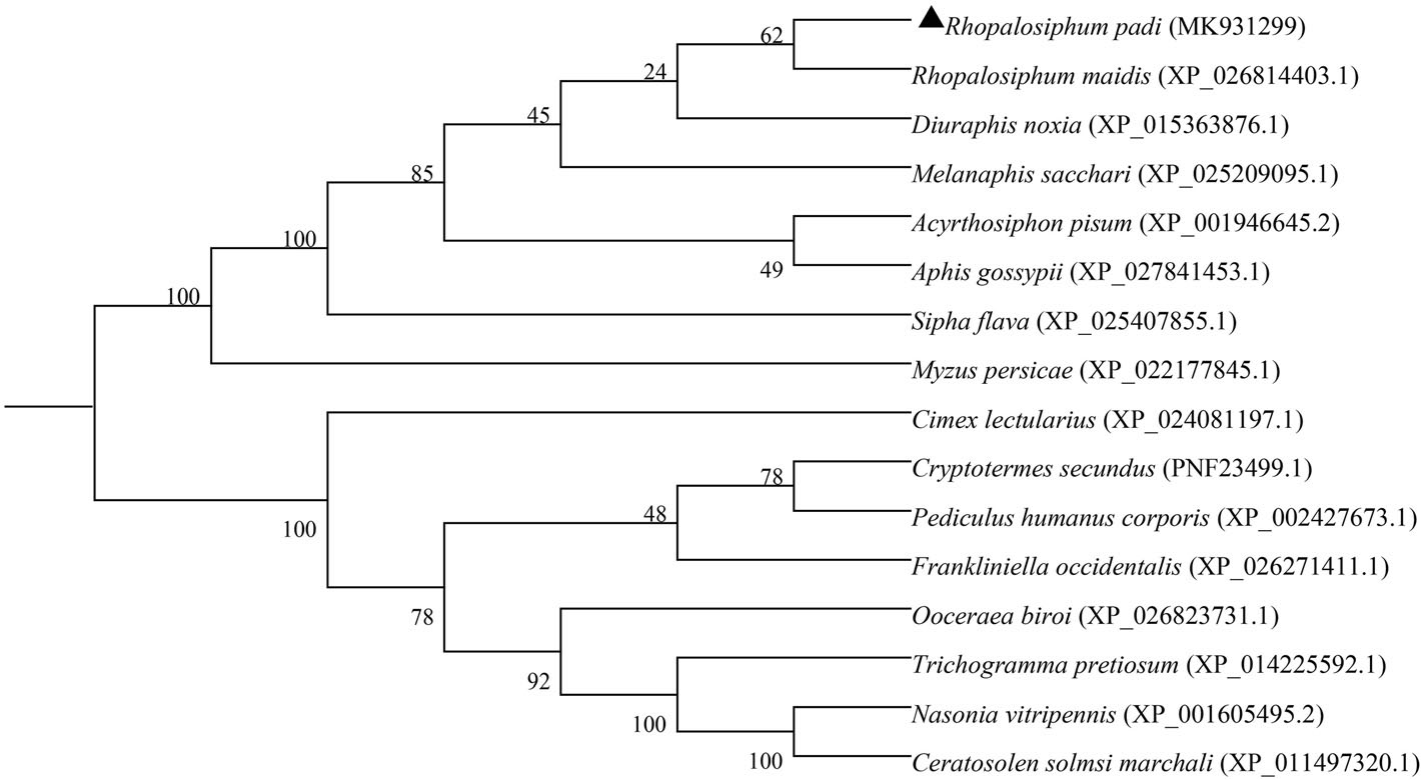
Phylogenetic relationship of *RpSTAT5B* with other insect STAT5Bs. Sequences were aligned using Clustal Omega and the neighbor-joining tree was constructed using MEGA6.0. Sequences used in the analysis are listed in Table S4

Multiple protein sequence alignment of *RpSTAT5B* with six STAT5B proteins from other insects was conducted. Structural analysis showed that the *RpSTAT5B* protein has the typical structural features of the STAT5B family (Fig. 5). Phylogenetic analysis indicated that the amino acid sequence of the *RpSTAT5B* protein showed high identity to that of the corresponding proteins from other aphids of Homoptera, particularly *Rhopalosiphum maidis* (Fig. 6). Both the RT-qPCR and transcriptome results showed that *RpSTAT5B* was upregulated in *R. padi* fed on WDV infected wheat (Fig. 4), suggesting that *RpSTAT5B* is critical to the response to feeding on infected wheat plants. However, the mechanism of *RpSTAT5B* in aphid immune defense remains to be further studied.

## Discussion

Our study investigated the effects of plant viruses on vector and non-vector insects at the transcriptional level. The number of DEGs was greater in the vector aphids (Sg-BYDV with 1525) than in the non-vector aphids (Sg-WDV, Rp-BYDV, and Rp-WDV with 494, 589 and 343, respectively). For all DEGs, the analysis showed that many of the DEGs were involved in development, growth and reproduction. The numbers of genes related to these processes were higher in the vectors (Sg-BYDV) than in the non-vectors (Sg-WDV, Rp-BYDV, and Rp-WDV).

For the GO analyses of all DEGs, the genes associated with the cuticles were mostly downregulated. Particularly in the vector aphids (Sg-BYDV), there were 16 genes associated with cuticles that were differentially expressed, among which 15 genes were downregulated. Moreover, fewer related DEGs were identified in the non-vectors, i.e., two DEGs in Sg-WDV, five DEGs in Rp-BYDV, and two DEGs in Rp-WDV. The cuticle is of great importance for the survival of insects, since it is the articulated exoskeleton that protects the body against the invasion of pathogens [28, 29]. The cuticle and its primary biopolymer components, cuticular proteins and chitin, of insects are periodically turned-over and new cuticle is secreted by the insect epidermis during ecdysis (molting) to accommodate the rapid growth and expansion of the body, and it is thought that temporally and spatially-dynamic epidermal expression of diverse cuticular and endocuticle proteins occurs to support the structure of different hard and soft cuticles of insect body parts during development [30]. The down-regulation of genes associated with the cuticles in aphids under wheat virus infection may signify a delay in molting or turnover of cuticle-associated proteins. One study reported a similar downregulation of six of seven cuticular proteins (CP) transcript sequences of *Plodia interpunctella* (Indian meal moth) 24 h after exposure to the baculovirus Plodia interpunctella Granulosis Virus (PiGV) [31]. The authors hypothesized that infection suppressed activities of cuticular proteins embedded in the peritrophic matrix, a structural barrier to pathogen attack [32, 33]. However, they alternatively offered the possibility that the pathogenic virus may also negatively affect molting.

Insects rely on their immune system to fight against pathogens [34]. After feeding on virus-infected wheat plants, the DEGs related to immunity in *S. graminum* and *R. padi* were upregulated, including the JAK-STAT signaling pathway, the lysosome, antigen processing and presentation, ubiquitin mediated proteolysis and the peroxisome. As shown in our results, the number of DEGs related to immunity in *S. graminum* exposed to BYDV was higher than that in *S. graminum* exposed to WDV and in *R. padi* exposed to BYDV and WDV. These results suggest that feeding on virus-infected plants has a greater effect on the immune system of vector insects than that of non-vector insects. The DEGs involved in the cytoskeleton were also differentially expressed, which may be related to the immune response [35]. There have been previous studies showing that viruses can interact with and reorganize host cytoskeleton components for intercellular trafficking and infection processes [18, 36–38]. In addition, the cytoskeleton is also commonly involved in the intracellular transport of viruses [39–42].

There were 3 DEGs upregulated in Sg-BYDV that were related to ubiquitin mediated proteolysis, which is important for insect defenses against pathogens [43]. In this pathway, we identified that UBE2D (ubiquitin-conjugating enzyme E2 D), BIRC2_3 (baculoviral IAP repeat-containing protein 2/3) and PIAS1 (E3 SUMO-protein ligase PIAS1) were upregulated. The E2 enzyme is the ubiquitin carrier protein or ubiquitin-conjugating enzyme that can transfer ubiquitin from E1 to the substrate [44]. Inhibitors of apoptosis proteins (IAPs), also known as baculovirus IAP repeat (BIR)-containing proteins (BIRCs), generally display anti-apoptotic properties when overexpressed [45]. PIAS1 has been recognized as a small ubiquitin-like modifier (SUMO) ligase [46].

Lysosome pathway is important in humoral immune response. We identified CTSB (cathepsin B), LGMN (legumain), LAMAN (lysosomal alpha-mannosidase), NPC2 (Niemann-Pick C2 protein) associated with lysosome were up-regulated in *S. graminum* exposed to BYDV; LAMAN was up-regulated in *S. graminum* and *R. padi* exposed to WDV. Cathepsins are proteases involved in protein degradation, apoptosis, and signaling, and they regulate viral infection and transmission [47–49]. In the green peach aphid, a lysosomal cathepsin B is upregulated following acquisition of the circulative-transmitted virus, *Potato leafroll virus* (PLRV), and together, the protein and virus colocalize at the cell membranes of midgut cells [49].

The JAK-STAT signaling pathway is important for innate immunity and antiviral responses in insects [50–53]. This pathway consists of the ligand unpaired (UPD), the receptor Domeless, JAK, STAT, protein inhibitor of activated STAT (PIAS), suppressor of cytokine signaling (SOCS), and signal transducing adaptor molecule (STAM) [43, 54, 55]. We found that PIAS1 (Sg55104) was up-regulated in the Sg-BYDV aphids; this gene encodes E3 SUMO-protein ligase PIAS1. A previous study has shown that PIAS1 is a specific inhibitor of Stat1-mediated gene activation [56]. In addition, STAT was up-regulated in the Rp-WDV aphids. JAK activation occurs upon ligand-mediated receptor multimerization, and the activated JAKs subsequently phosphorylate additional targets, including both the receptors and the major substrates, STATs. Phosphorylated STATs enter the nucleus by a mechanism that is dependent on importin a-5 (also called nucleoprotein interactor 1) and the Ran nuclear import pathway. Once in the nucleus, dimerized STATs bind specific regulatory sequences to activate or repress transcription of target genes (Fig. S14) [57].

**Fig. 7:**
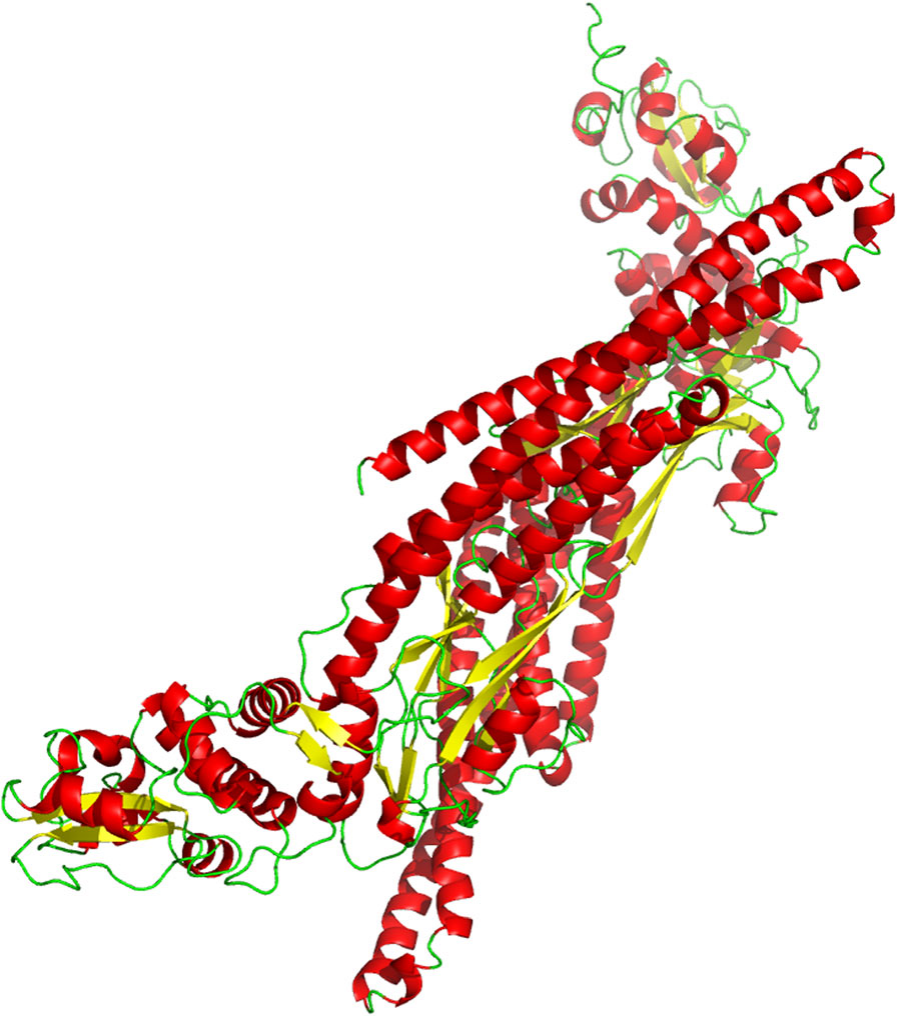
Predicted protein structure of *RpSTAT5B*

STAT5B was the only related gene that was differentially expressed in *R. padi* fed on WDV infected wheat. *RpSTAT5B* possesses the SH2 (Src-homology Domain), STAT5_CCD (Coiled-coiled Domain), and the STAT_DBD (STAT_bind) conserved domains, which are the characteristic conserved domains of STAT5 (Fig. 5) [58–62]. For prediction of its three-dimensional structure, the SWISS-MODEL template library (SMTL version 2019-06-27, PDB release 2019-06-21) was searched with BLAST and HHBlits for evolutionary related structures matching *RpSTAT5B*. The predicted three-dimensional structure of *RpSTAT5B* is shown in Fig. 7, and its identity with the template (SMTL ID: 1y1u.1.A) is 45.47%. Overall, the identification of *RpSTAT5B*, and its increased expression in *R. padi* fed on WDV infected wheat, suggests it may fulfill a specific physiological role. Future studies are needed to understand the function of *RpSTAT5B* in *R. padi* fed on WDV infected wheat.

Insect pests and viral diseases are important factors affecting wheat yield. In this study, we analyzed the response of vector/non-vector aphids to wheat virus at the transcriptional level. Among all DEGs analyzed, we identified several genes associated with immunity, growth, development, reproduction and the cuticle of aphids. The results revealed that the number of DEGs and the functions related to the DEGs were both higher in vector aphids than that in non-vector aphids. This may imply that wheat viruses have a larger impact on vector than non-vector aphids. Exposure to the virus activated the immunity response of the aphids, particularly vector aphids. These results will provide a reference for investigation of new methods to improve the efficiency of pest control, including prokaryotic expression and gene silencing.

## Methods

### Insect rearing and plant infection by the viruses

Aphids from each of the species *S. graminum* and *R. padi* was collected from Yangpingguan (34.85°N, 105.63°E), Shaanxi Province, and they were maintained on wheat seedlings in growth chambers kept at 20 ± 1 °C, 65% ± 5% relative humidity and a photoperiod of 16: 8 h (L: D). Aphids used in the study were parthenogenetic descendants from a single isolated female. BYDV (isolate BYDV-GAV) and WDV were collected from Yangling (34.28°N, 108.22°E) and Hancheng (35.47°N, 110.45°E), Shaanxi Province, China, from winter wheat plants. The viruses were identified using RT-PCR and agarose gel electrophoresis using the methods briefly described below.

We isolated total RNA from the wheat using TRIzol reagent (TaKaRa, Japan) following the manufacturers’ instructions to test whether wheat was infected by BYDV. The cDNA was synthesized using PrimeScript RT Master Mix (TaKaRa, Japan). We isolated total gDNA from the wheat using DNAsecure Plant Kit (TIANGEN, China) following the manufacturers’ instructions to test whether wheat was infected by WDV. Then PCR was performed; the primers used for the virus identification are shown in Table S1. The PCRs consisted of the following: 6.5 μl of Reaction Mix (TIANGEN, China), 1 μl of each forward and reverse primer, 1 μl of cDNA/gDNA and 4.5 μl of ddH2O. The cycling conditions were 94 °C for 3 min; 35 cycles of 94 °C for 30 s, 55 °C for 30 s, and 72 °C for 1 min; and finally, 72 °C for 5 min. Then, agarose gel electrophoresis was performed, and we sequenced the PCR products to confirm that the wheat was infected by BYDV or WDV. The detailed methods of the BYDV-infected and WDV-infected plant treatments, as transmitted by the leafhopper and *S. graminum*, respectively, have been previously described as follows [63]. Wheat plants at the second leaf stage were first covered with transparent, plastic, tube-shaped cages (30 cm in height, 13.5 cm in diameter, and with a mesh screen cover on the top), and then 5 late instar nymphs of the vector were introduced into the cage and allowed to feed on the leaves. All the nymphs were removed after 5 days, and the treated plants were maintained under the growth chamber described as above.

### Feeding assays and RNA isolation

The young first instar nymphs of *S. graminum* and *R. padi* were fed on BYDV-infected, WDV-infected, and healthy wheat plants to achieve six experimental treatments: the vector treatment was defined as *S. graminum* fed on BYDV-infected wheat (Sg-BYDV); the non-vector treatment was defined as *S. graminum* fed on WDV-infected wheat and *R. padi* fed on BYDV-GAV-infected, WDV-infected wheat (Sg-WDV, Rp-BYDV, Rp-WDV); and the control treatments were *S. graminum* and *R. padi* fed on healthy wheat seedlings (Sg-ck, Rp-ck).

Three biological replications of each treatment were conducted and each replicate contained 20 individual aphids. For each treatment-replicate, they were maintained in a growth chamber under the same environmental conditions as described above. We collected 20 adult aphids into a 1.5 ml tube, and they were immediately flash frozen in liquid nitrogen and stored at − 80 °C. Total RNA was extracted from each sample using TRIzol reagent (TaKaRa, Japan) following the manufacturers’ instructions. RNA degradation and contamination were monitored on 1% agarose gels. RNA purity was checked using the NanoPhotometer® spectrophotometer (IMPLEN, CA, USA). RNA concentration was measured using a Qubit® RNA Assay Kit in a Qubit®2.0 Fluorometer (Life Technologies, CA, USA). RNA integrity was assessed using the RNA Nano 6000 Assay Kit of the Agilent Bioanalyzer 2100 system (Agilent Technologies, CA, USA).

### Transcriptome sequencing and analysis

A total amount of 3 μg of RNA per sample was used as input material for the RNA sample preparations. Sequencing libraries were generated using the NEBNext® Ultra™ RNA Library Prep Kit for Illumina® (NEB, USA) following the manufacturer’s recommendations. Briefly, mRNA was purified from the total RNA using poly-T oligo-attached magnetic beads. Fragmentation was carried out, and the first and second strands were synthesized. The purified and adaptor-ligated cDNA was subjected to PCR amplification. Finally, the PCR products were purified (AMPure XP system), and the library quality was assessed on the Agilent Bioanalyzer 2100 system.

The clustering of the index-coded samples was performed on a cBot Cluster Generation System using a TruSeq PE Cluster Kit v3-cBot-HS (Illumina) according to the manufacturer’s instructions. After cluster generation, the library preparations were sequenced on an Illumina Hiseq 2000 platform and paired-end reads were generated. Clean data were obtained by removing reads containing adapters, reads containing poly-N and low-quality reads. At the same time, the Q20, Q30, GC-content and sequence duplication level of the clean data were calculated. All of the downstream analyses were based on clean data with high quality. Transcriptome assembly was accomplished using Trinity (r20131110) with min_kmer_cov set to 2 by default and all other parameters set to default [64].

Differential expression analysis of the two groups was performed using the DESeq R package (1.10.1). DESeq provides statistical routines for determining differential expression of digital gene expression data using a model based on the negative binomial distribution. The resulting *P* values were adjusted using the Benjamini and Hochberg’s approach for controlling the false discovery rate. Genes with an adjusted *P*-value < 0.05 found by DESeq were considered differentially expressed.

Gene ontology (GO) enrichment analysis of the differentially expressed genes (DEGs) was implemented by the topGO R packages based on the Kolmogorov–Smirnov test. We used KOBAS software to test the statistical enrichment of the differentially expressed genes in the KEGG pathways. Genes with similar expression patterns are usually functionally related. Cluster 3.0 software was used to analyze differentially expressed genes by hierarchical clustering with Euclidean distance as the distance matrix. The clustering results were displayed by Java Treeview.

### RT-qPCR validation

To validate differential expression in response to the viruses, 13 transcript sequences were selected in *S. graminum* and *R. padi* and then compared with the expression levels between the virus-exposed and non-virus-exposed aphids. Actin was used as an internal reference gene to normalize the expression level. The primers were designed using Primer Premier 5.0 software and are shown in Table S2. The total RNA extraction and cDNA synthesis methods were performed as described above. Total RNA was isolated from three biological replicates, and each targeted gene included three technical replicates.

The reactions of real-time RT-qPCR were performed in 25 μl volumes using TB Green Premix Ex Taq (TaKaRa, Japan) with 12.5 μl of TB Green Premix Ex Taq II, 1 μl of each forward and reverse primer (10 μM), 0.5 μl of ROX Reference Dye II, 2 μl of cDNA and 8 μl of nuclease free water. The cycling parameters were as follows: 95 °C for 30 s and 40 cycles of 95 °C for 5 s and 60 °C for 30 s. A standard curve experiment was set up by performing a dilution series with 5^− 1^ dilution times and 5 dilution points to evaluate the PCR efficiency (E). The Q-PCR was performed on a QuantStudio 3 Real-Time PCR System (Applied Biosystems), and QuantStudio™ Design and Analysis Software was used to analyze the PCR assays. The relative quantitative data analysis was performed using the 2^-△△ct^ method.

### Cloning and characterization of the STAT5B

STAT5B (signal transducer and activator of transcription 5B) was the only relevant gene that was differentially expressed in *R. padi* fed on WDV infected wheat. To understand the mechanism of STAT5B in the insect immune defense response, we designed primers using Primer Premier 5.0 to clone STAT5B based on the sequence found in the transcriptome (Table S3). PCR analyses were conducted on a PCR Amplifier (purchased from Eppendorf, Yangling, China) using Tks Gflex™ DNA Polymerase (purchased from TaKaRa, Yangling, China). Cloned products were validated by RT-PCR and sequencing. Multiple sequence alignments were performed using Clustal W and DNAMAN. The phylogenetic tree was generated in Mega 6 using the Neighbor-Joining method. All sequences used in the analysis are listed in Table S4. The protein structure model was predicted using WISS-MODEL (https://swissmodel.expasy.org/) and visualized in Pymol (v1.3r1).

## Supplementary information

**Additional file 1 Figure S1**. GO enrichment of (A) *S. graminum*, (B) *R. padi.* **Figure S2**. GO enrichment analysis of DEGs of *S. graminum* fed on BYDV-infected wheat. **Figure S3**. GO enrichment analysis of DEGs of *S. graminum* fed on WDV-infected wheat. **Figure S4**. GO enrichment analysis of DEGs of *R. padi* fed on BYDV-infected wheat. **Figure S5**. GO enrichment analysis of DEGs of *R. padi* fed on WDV-infected wheat. **Figure S6**. COG enrichment analysis of DEGs of *S. graminum* fed on BYDV-infected wheat. **Figure S7**. COG enrichment analysis of DEGs of *S. graminum* fed on WDV-infected wheat. **Figure S8**. COG enrichment analysis of DEGs of *R. padi* fed on BYDV-infected wheat. **Figure S9**. COG enrichment analysis of DEGs of *R. padi* fed on WDV-infected wheat. **Figure S10** KEGG pathway analysis of DEGs of *S. graminum* fed on BYDV-infected wheat. **Figure S11**. KEGG pathway analysis of DEGs of *S. graminum* fed on WDV-infected wheat. **Figure S12**. KEGG pathway analysis of DEGs of *R. padi* fed on BYDV-infected wheat. **Figure S13**. KEGG pathway analysis of DEGs of *R. padi* fed on WDV-infected wheat. **Figure S14** JAK/STAT signaling pathway.

**Additional file 2 Table S1**. Primers of the genes used for RT-PCR. **Table S2**. Primers of the genes used for RT-qPCR. **Table S3**. Primers of STAT5B. **Table S4** Sequences used in the STAT5B analysis. **Table S5**. DEGs related to development, reproduction and growth of Sg-BYDV, Sg-WDV, Rp-BYDV, and Rp-WDV.

## Data Availability

Transcriptome data are available at NCBI with raw sequencing reads deposited in the Sequence Read Archive (accession # PRJNA490258). We have submitted the sequence of *RpSTAT5B* to NCBI GeneBank database and the accession number is MK931299.

## Abbreviations

BYDV: *Barley yellow dwarf virus*
WDV: *Wheat dwarf virus*
*S. graminum*: *Schizaphis graminum*
*R. padi*: *Rhopalosiphum padi*
DEGs: Differentially expressed genes
Sg-BYDV: *Schizaphis graminum* feeding on wheat infected by BYDV
Sg-WDV: *Schizaphis graminum* feeding on wheat infected by WDV
Rp-BYDV: *Rhopalosiphum padi* feeding on wheat infected by BYDV
Rp-WDV: *Rhopalosiphum padi* feeding on wheat infected by WDV
STAT5B: Signal transducer and activator of transcription 5B
TSWV: *Tomato spotted wilt virus*
SRBSDV: *Southern rice black-streaked dwarf virus*
WBPH: White backed planthopper
RBSDV: *Rice black streaked dwarf virus*
RT-qPCR: Quantitative real-time PCR
GO: Gene ontology
KEGG: Kyoto Encyclopedia of Genes and Genomes
