# Supplementary material for: Transcriptome response comparison between vector and non-vector aphids after feeding on virus-infected wheat plants
Source: BMC Genomics. 2020 Sep 15;21:638. doi: 10.1186/s12864-020-07057-0 (PMC7493910; doi:10.1186/s12864-020-07057-0)
Supplement: Supplementary file 2 — Additional file 2 Table S1. Primers of the genes used for RT-PCR. Table S2. Primers of the genes used for RT-qPCR. Table S3. Primers of STAT5B. Table S4 Sequences used in the STAT5B analysis. Table S5. DEGs related to development, reproduction and growth of Sg-BYDV, Sg-WDV, Rp-BYDV, and Rp-WDV. [file 12864_2020_7057_MOESM2_ESM.docx]

Table S1 Primers of the genes used for RT-PCR

| Primer | forward | reverse |
| --- | --- | --- |
| BYDV-GAV  WDV | ATGAATTCAGTAGGCCGTAGA  CGACTACGCCTGGCGAACATTTG | CTATTTGGGAGTCATGTTGGC  TCTGGCATTGCC TGTTTCGG |

Table S2 primers of the genes used for RT-qPCR

| Gene ID | forward | reverse |
| --- | --- | --- |
| Sg30601  S35675  Sg35800  Sg36590  Sg40066  Sg43786  Sg48920  Sg51592  Sg53134  Sg56027  Rp22910  Rp22931  Rp46740  Actin | TTCAGGACGAAGATGTGAAAACTC  GGTGAATCGGCTGAGGACA  CTGATTCTGATACTCTTCGGGTAGG  ACGCCTCTTTCATCTGTTATTTCC  GATCGGTTATTATTGTGGCTTGG  CTCGTTCAAGTGGCACAACAC  CATTCGTGACCACTCTGAACCT  TCAACCTGCTCCAACCTCAA  AGGACAAGCGGGAAGACAAG  TGTGGAAGTCATCTGCTGGAGT  AGCCAAGTATCGTTTGCCATTT  TTCCTGTGCCTGGCTTTTG  GATCGGTTATTATTGTGGCTTGGT  CGTTACCAACTGGGACGATATG | GGCCGTAATGGATCTGGAAA   \| CCATCTTGCGGTGTTTCTCTTT \| \| --- \|   CCAGAAGTTACCGTTTCACCAAG  ACGCCTCTTTCATCTGTTATTTCC  CGGTAGGAGATGGATGAAAAGG  CAACTACCAGAAACAGACCATCC  TTCTGCTGTTCGTGTTGCTGT  TTTACCACAGTCGATACTCATTCCA  GCGGTCGTTTCATTGGTAGAG  CGAGAGCCCCATCTTTGTTC  GGAAGAAGTTTTGTGCCTGCTT  GTTTCTCGTTTCCTGTTGGTCTTT  GGAAGGAGGTGGATGAAAAGG  GGGTTCAATGGAGCTTCTGTTA |

Table S3 primers of STAT5B

| using | forward | reverse |
| --- | --- | --- |
| clone  q-PCR | AGTACAGGCATTCCTAAATCCCGTCA  GTGAGCATTGCCGTTGGAT | CGCACTTAAAGCCCTTCCC  GTTTTGTAGCAGTTCCTCGTTGTTT |

Table S4 Sequences used in STAT5B analysis

| Source | Accession number |
| --- | --- |
| *Acyrthosiphon pisum*  *Melanaphis sacchari*  *Rhopalosiphum maidis*  *Sipha flava*  *Aphis gossypii*  *S. flava*  *Diuraphis noxia*  *Myzus persicae*  *Cimex lectularius*  *Cryptotermes secundus*  *Pediculus humanus corporis*  *Frankliniella occidentalis*  *Ooceraea biroi*  *Trichogramma pretiosum*  *Nasonia vitripennis*  *Ceratosolen solmsi marchali* | XP_008188159.1  XP_025209095.1  XP_026814403.1  XP_025407855.1  XP_027841453.1  XP_025407854.1  XP_015363876.1  XP_022177845.1  XP_024081197.1  PNF23499.1  XP_002427673.1  XP_026271411.1  XP_026823731.1  XP_014225592.1  XP_001605495.2  XP_011497320.1 |

Table S5 DEGs related to development, reproductive and growth in Sg-BYDV, Sg-WDV, Rp-BYDV, and Rp-WDV.

|  | **Gene ID** | **log2FC** | **FDR** |
| --- | --- | --- | --- |
| Sg-BYDV | Sg35800  Sg53861  Sg46070  Sg43842  Sg54544  Sg53134  Sg51592  Sg36590  Sg35675  Sg43786  Sg58294  Sg56027  Sg48920  Sg54570  Sg44087  Sg55529  Sg46870  Sg49676  Sg54713  Sg56315  Sg53905  Sg51586  Sg55291  Sg55696  Sg55548  Sg50416  Sg35893  Sg44535 | -3.159  -1.740  1.584  Inf  6.229  1.098  -1.401  -1.189  -1.645  1.019  Inf  -1.347  -1.538  -3.344  1.816  1.535  -1.038  -1.527  Inf  -4.338  Inf  -1.701  1.164  -1.143  -1.399  5.042  Inf  1.727 | 0.001974  0.00481  2.44E-06  1.88E-05  6.82E-06  3.88E-08  0.000782  0.001241  3.44E-11  6.09E-06  9.67E-06  0.000376  3.20E-10  4.09E-18  1.23E-10  2.22E-12  2.56E-07  0.008734  9.26E-07  2.10E-23  1.21E-07  4.91E-06  8.45E-06  1.61E-06  4.24E-12  0.001199  1.74E-07  1.79E-06 |
| Sg-WDV | Sg35800  Sg39253  Sg26680  Sg47610  Sg17266  Sg54570  Sg41903  Sg47806  Sg55089  Sg58699  Sg53905  Sg55968  Sg30601 | -3.848  -4.216  Inf  2.581  -6.149  -3.133  Inf  -3.104  5.629  -4.283  Inf  -5.527  -2.095 | 0.000454  5.95E-06  0.002425  0.004509  6.05E-10  6.67E-08  0.004384  0.001323  0.001958  0.008436  9.09E-09  6.80E-19  1.07E-10 |
| Rp-BYDV | Rp22931  Rp44751  Rp23660  Rp29892  Rp46916  Rp30324  Rp38790  Rp52411  Rp39012  Rp39650  Rp47650  Rp35438  Rp50802 | 3.899  2.091  -3.126  -4.719  -2.909  2.766  1.806  2.329  3.927  1.839  1.342  -3.579  -3.857 | 1.19E-12  0.007355  3.80E-08  2.19E-05  8.76E-09  1.58E-06  0.002409  0.003514  3.62E-13  0.000133  0.008336  0.000907  0.000136 |
| Rp-WDV | Rp49333  Rp48839  Rp35438 | 2.163  2.603  -5.379 | 0.006445  0.001872  7.04E-07 |
