# Supplementary material for: Transcriptome response comparison between vector and non-vector aphids after feeding on virus-infected wheat plants
Source: BMC Genomics. 2020 Sep 15;21:638. doi: 10.1186/s12864-020-07057-0 (PMC7493910; doi:10.1186/s12864-020-07057-0)
Supplement: Supplementary file 1 — Additional file 1 Figure S1. GO enrichment of (A) S. graminum, (B) R. padi. Figure S2. GO enrichment analysis of DEGs of S. graminum fed on BYDV-infected wheat. Figure S3. GO enrichment analysis of DEGs of S. graminum fed on WDV-infected wheat. Figure S4. GO enrichment analysis of DEGs of R. padi fed on BYDV-infected wheat. Figure S5. GO enrichment analysis of DEGs of R. padi fed on WDV-infected wheat. Figure S6. COG enrichment analysis of DEGs of S. graminum fed on BYDV-infected wheat. Figure S7. COG enrichment analysis of DEGs of S. graminum fed on WDV-infected wheat. Figure S8. COG enrichment analysis of DEGs of R. padi fed on BYDV-infected wheat. Figure S9. COG enrichment analysis of DEGs of R. padi fed on WDV-infected wheat. Figure S10 KEGG pathway analysis of DEGs of S. graminum fed on BYDV-infected wheat. Figure S11. KEGG pathway analysis of DEGs of S. graminum fed on WDV-infected wheat. Figure S12. KEGG pathway analysis of DEGs of R. padi fed on BYDV-infected wheat. Figure S13. KEGG pathway analysis of DEGs of R. padi fed on WDV-infected wheat. Figure S14 JAK/STAT signaling pathway. [file 12864_2020_7057_MOESM1_ESM.pdf]

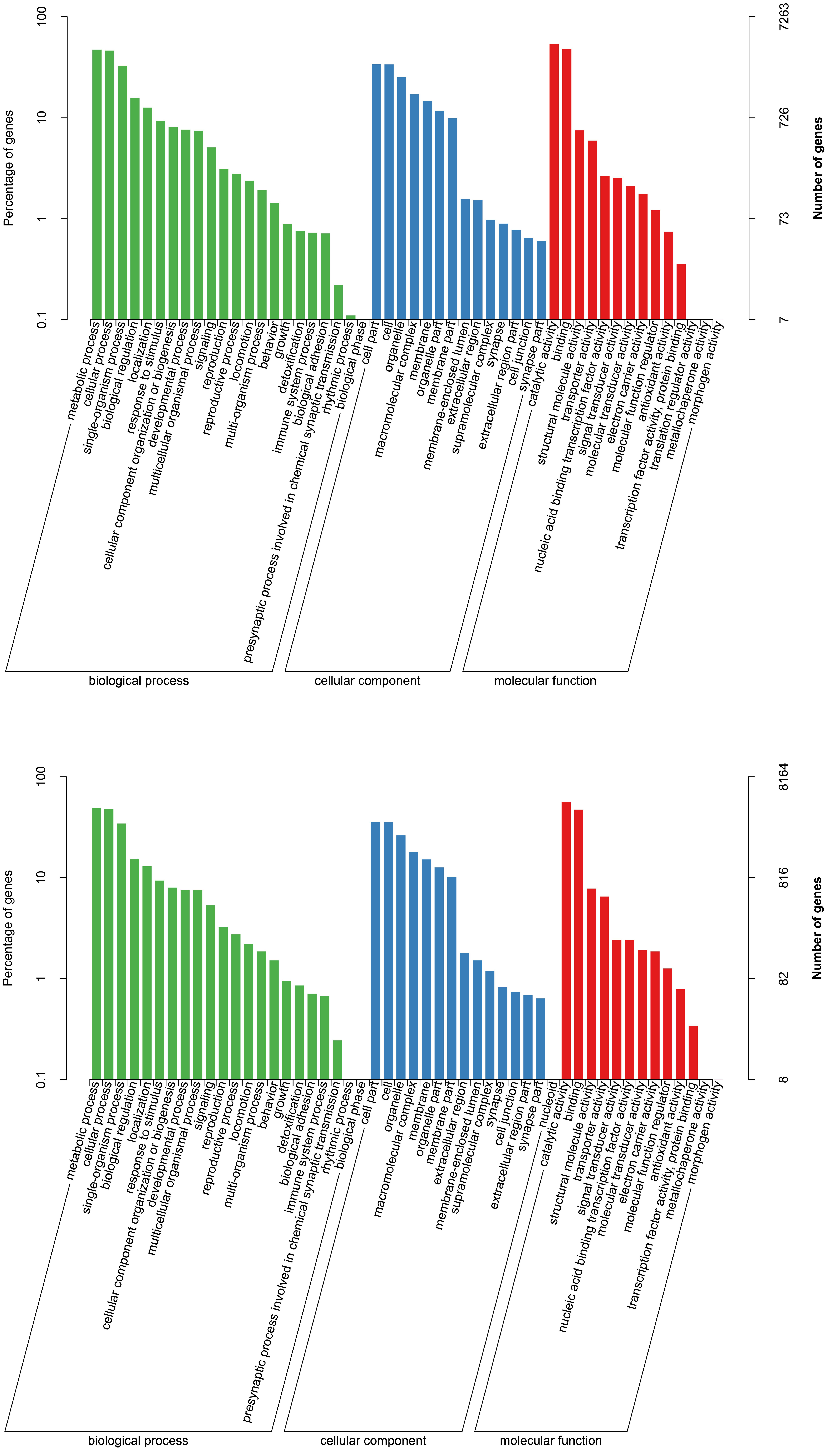

Fig. S1 GO enrichment of (A) *S. graminum*, (B) *R. padi*.

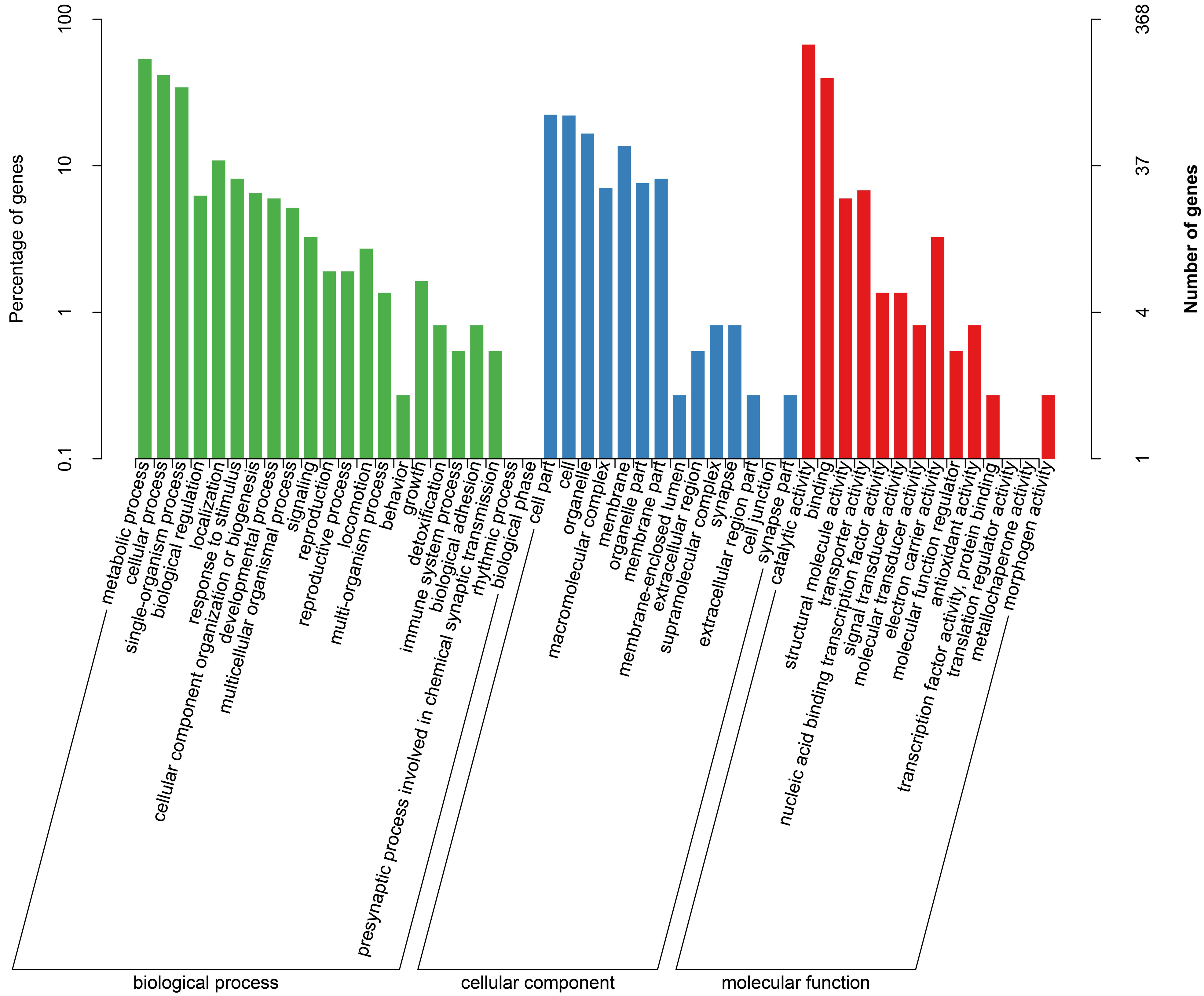

Fig. S2 GO enrichment analysis of DEGs of *S. graminum* fed on BYDV-infected wheat.

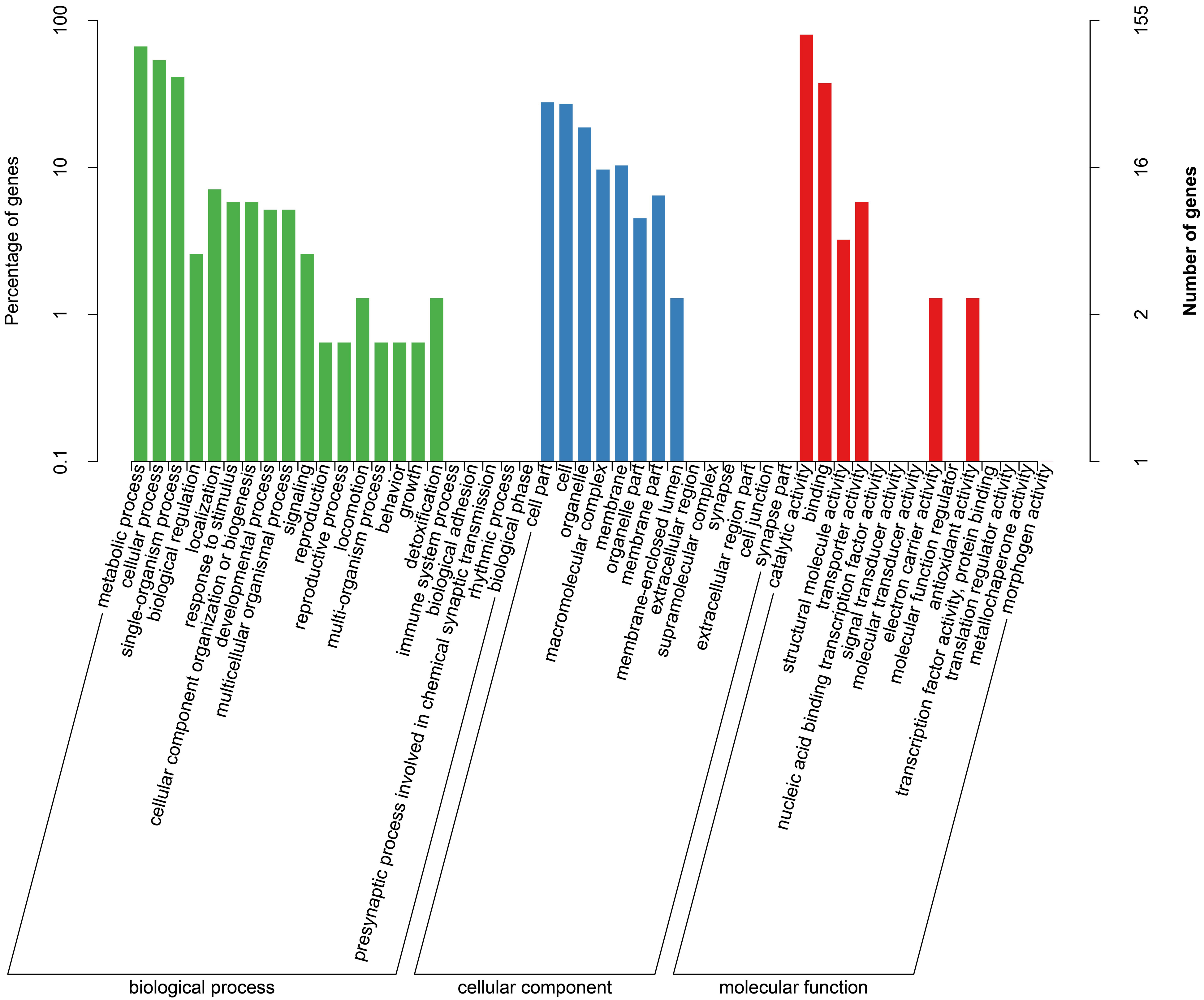

Fig. S3 GO enrichment analysis of DEGs of *S. graminum* fed on WDV-infected wheat.

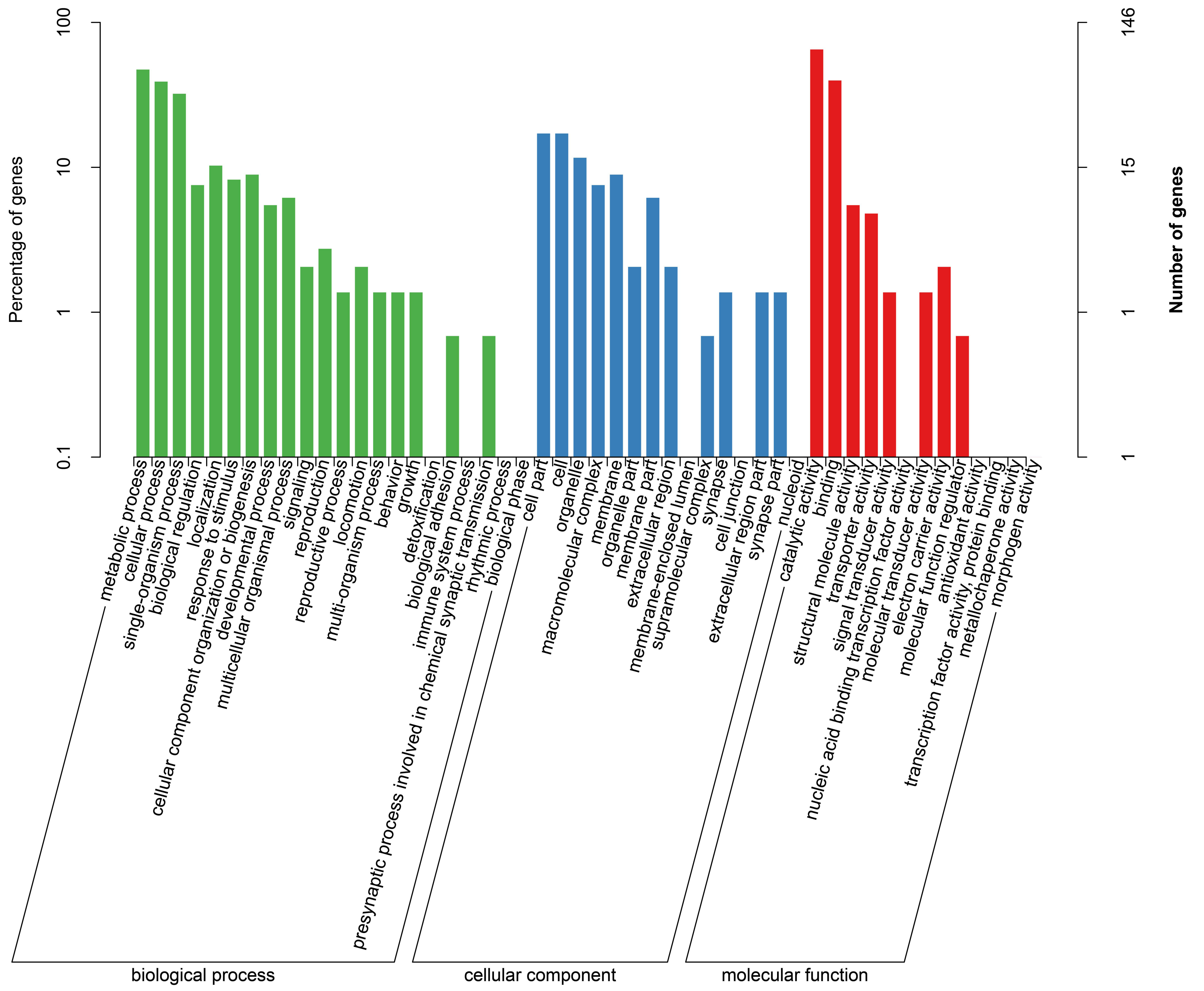

Fig. S4 GO enrichment analysis of DEGs of *R. padi* fed on BYDV-infected wheat.

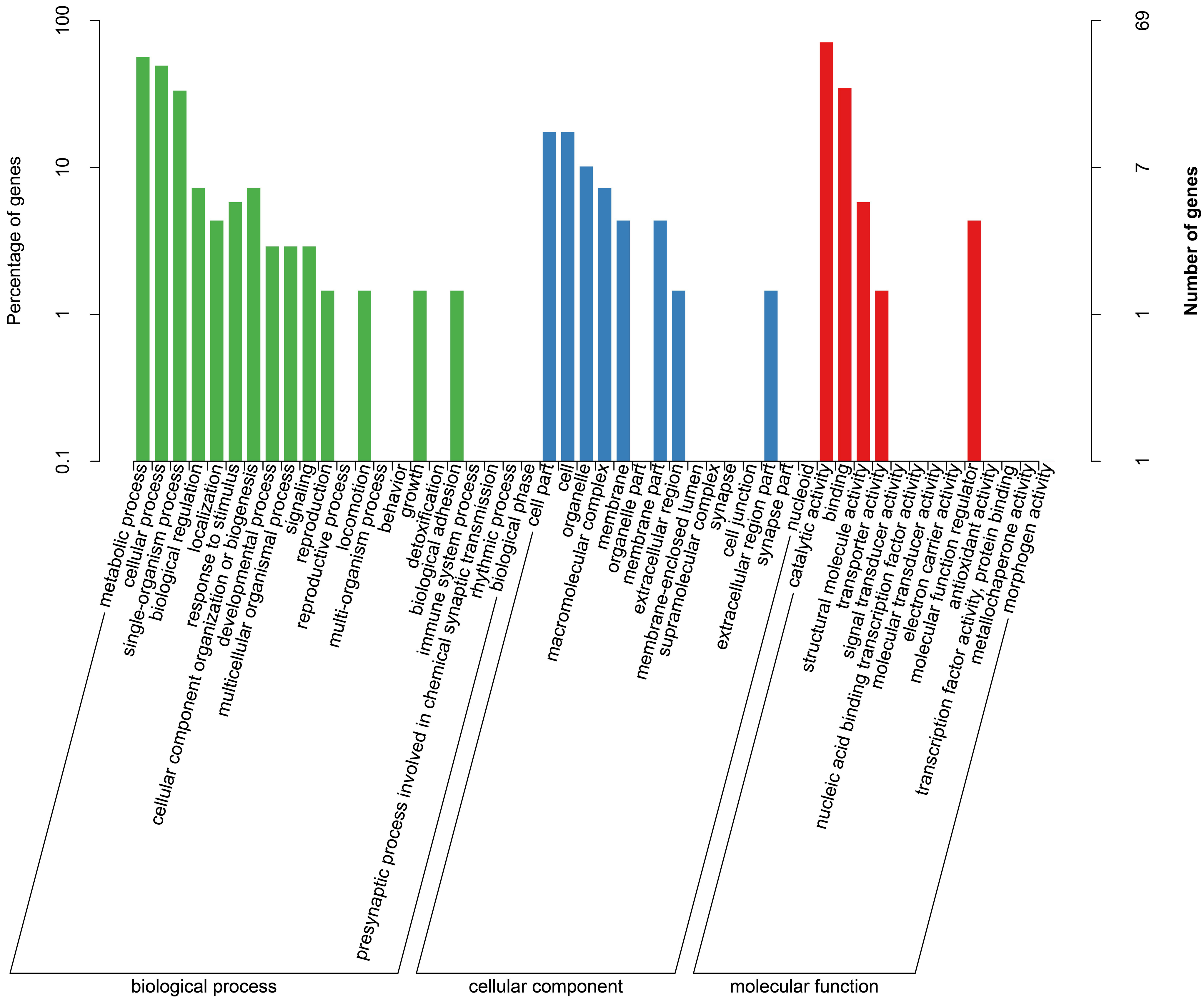

Fig. S5 GO enrichment analysis of DEGs of *R. padi* fed on WDV-infected wheat.

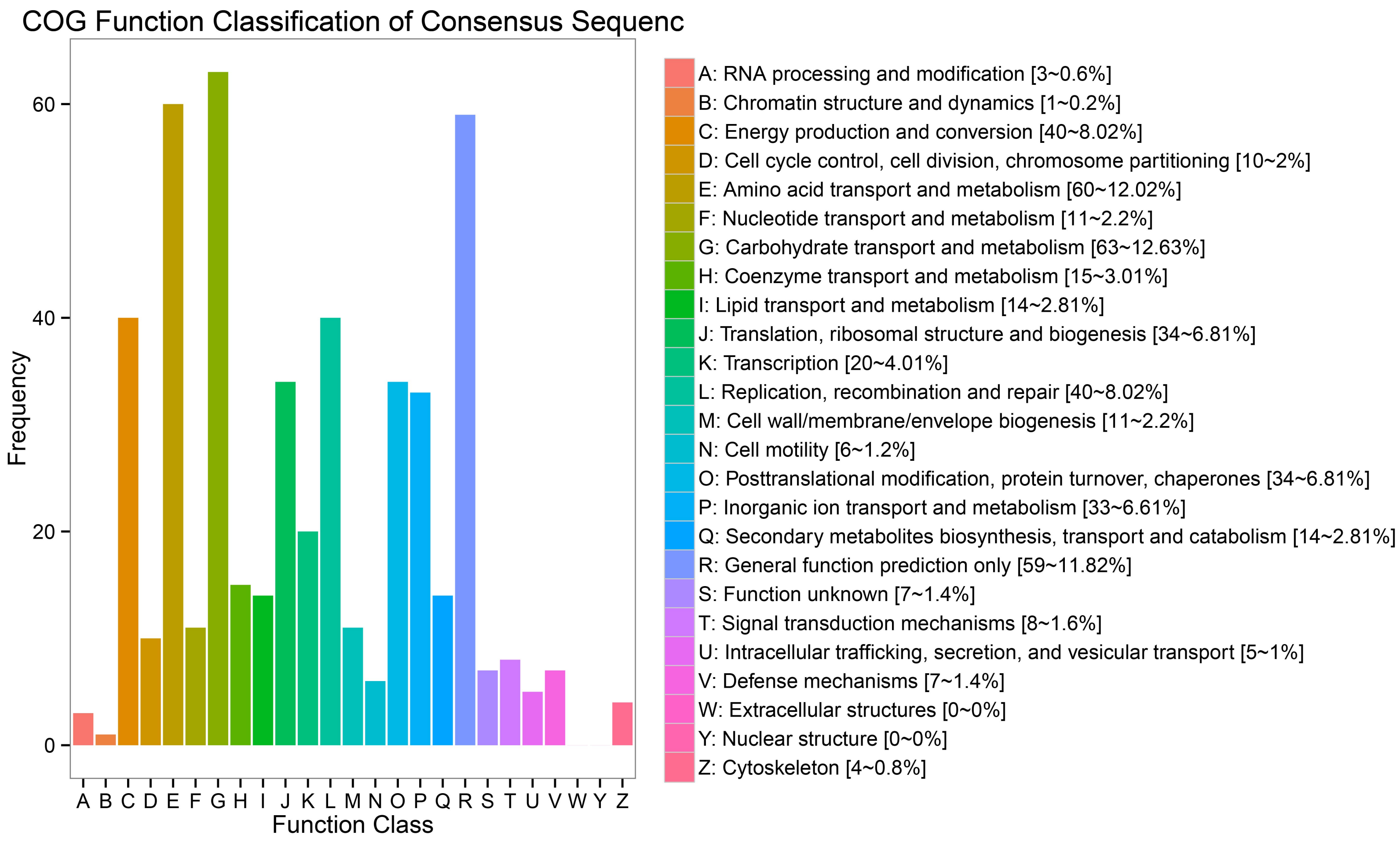

Fig. S6 COG enrichment analysis of DEGs of *S. graminum* fed on BYDV-infected wheat.

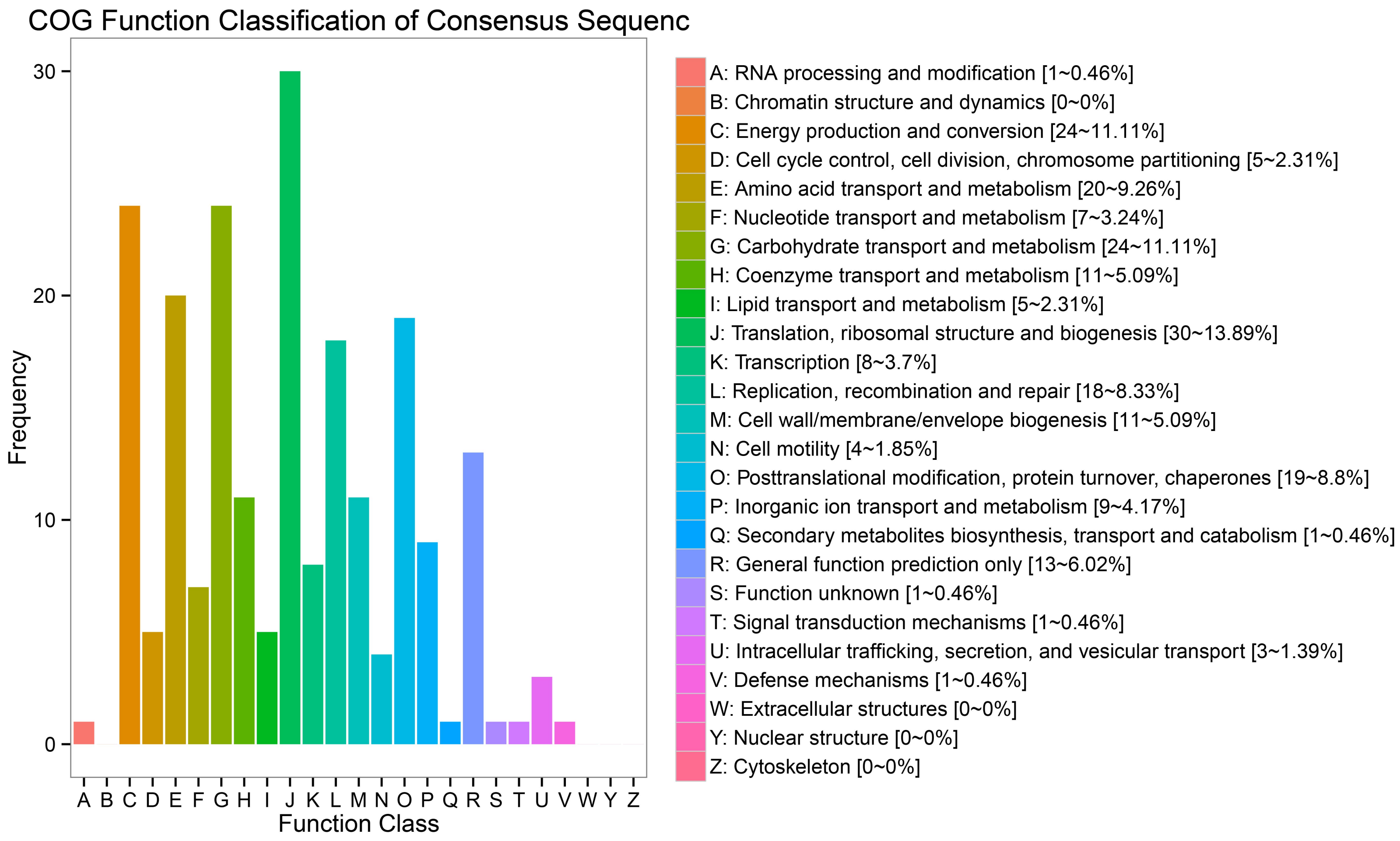

Fig. S7 COG enrichment analysis of DEGs of *S. graminum* fed on WDV-infected wheat.

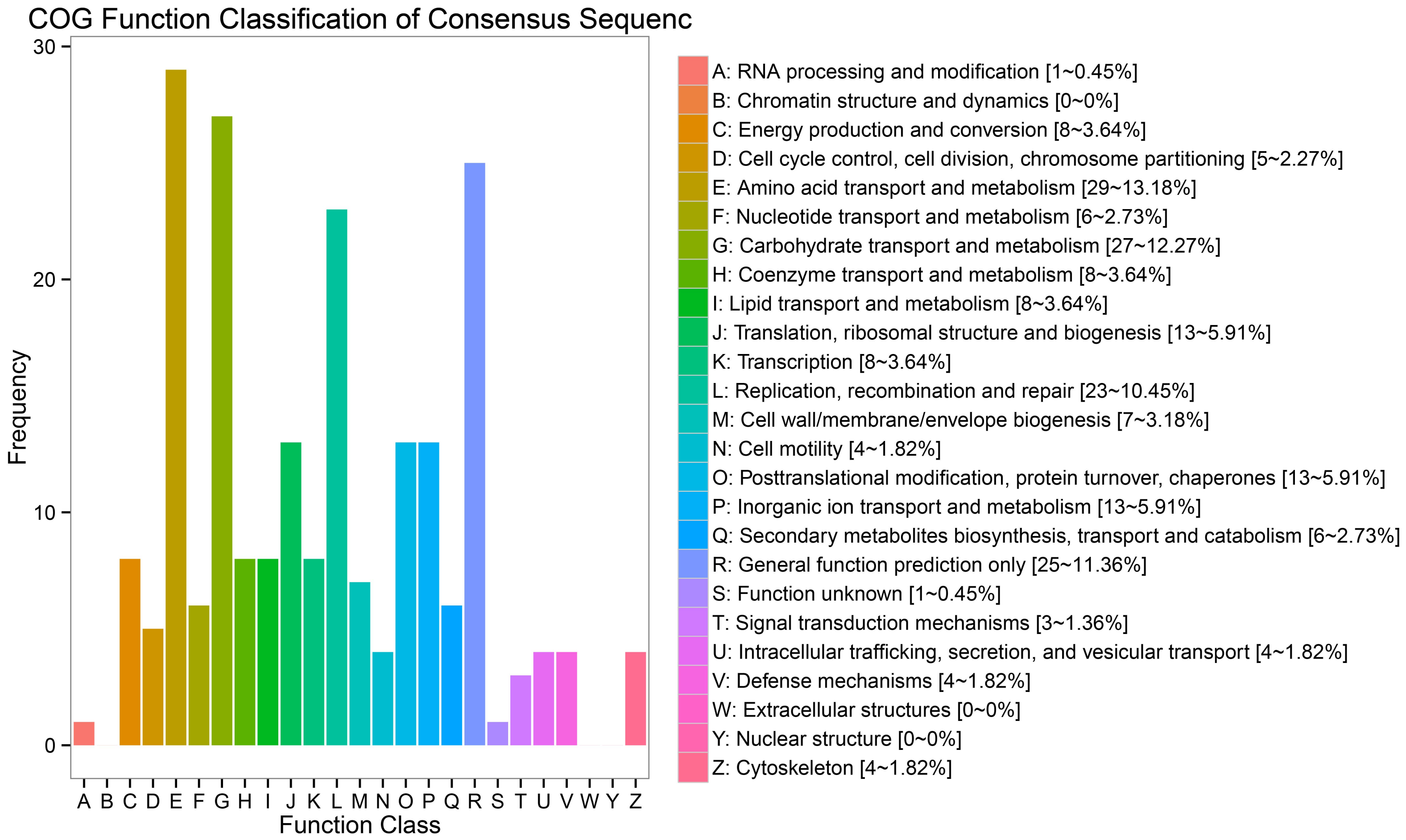

Fig. S8 COG enrichment analysis of DEGs of *R. padi* fed on BYDV-infected wheat.

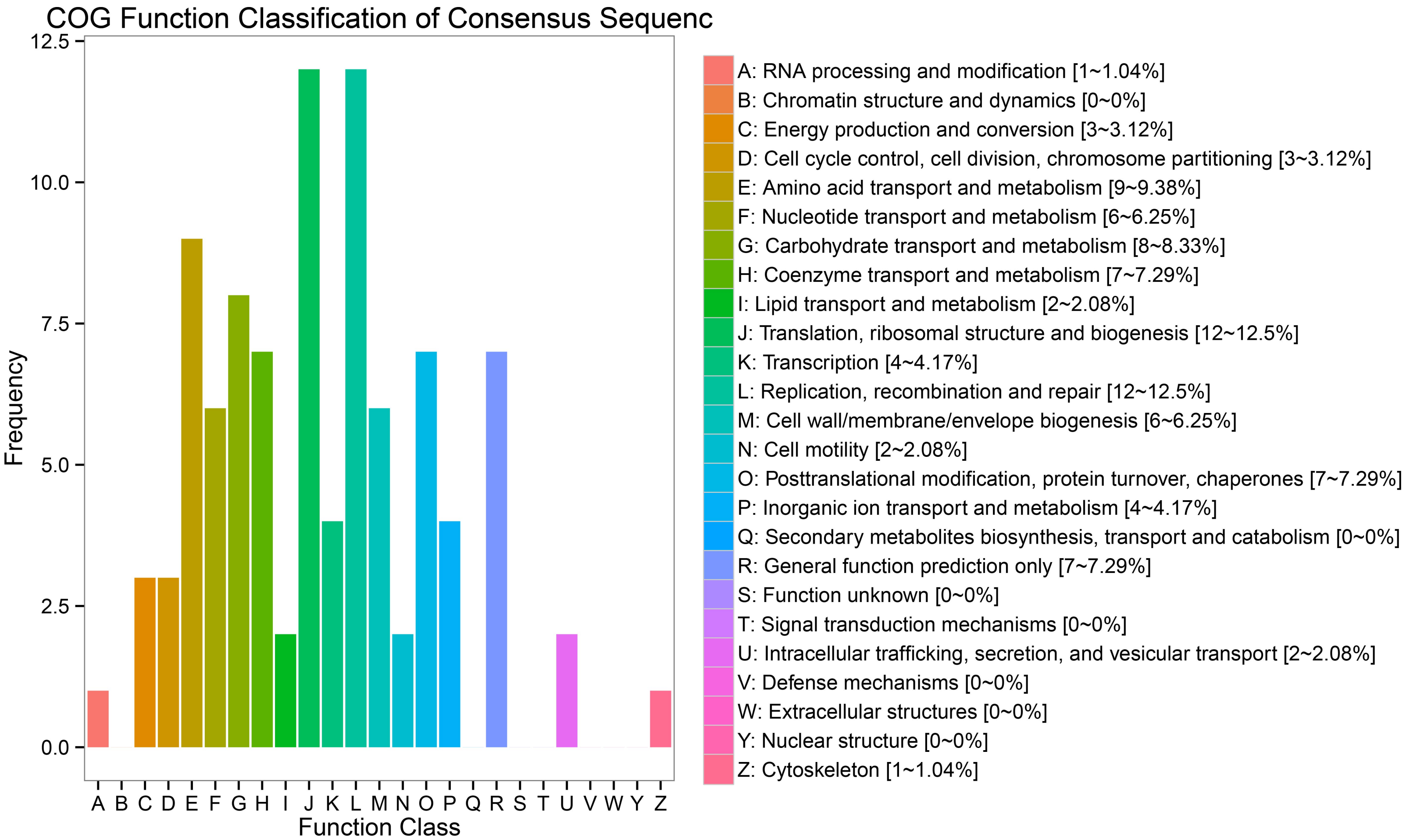

Fig. S9 COG enrichment analysis of DEGs of *R. padi* fed on WDV-infected wheat.

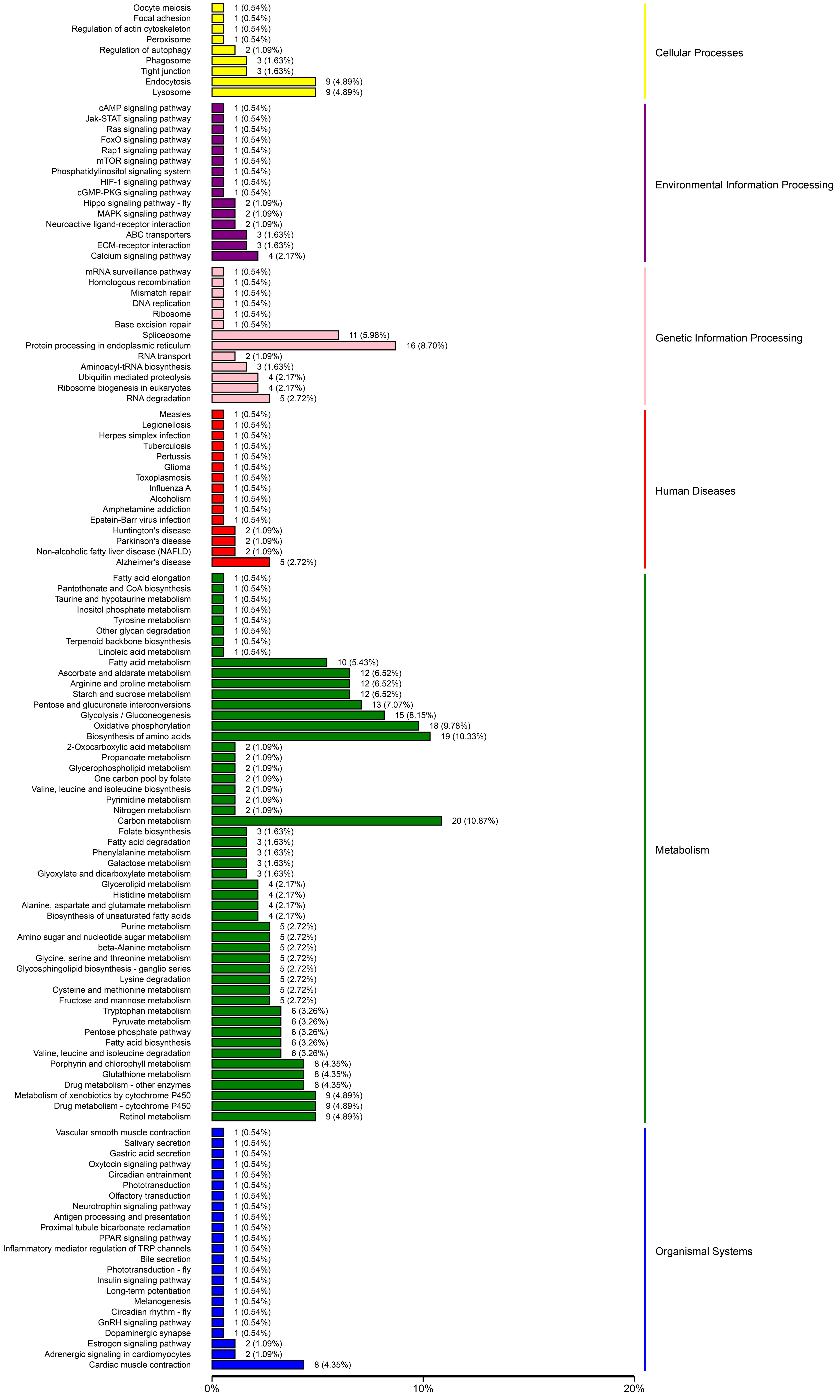

Fig. S10 KEGG pathway analysis of DEGs of *S. graminum* fed on BYDV-infected wheat.

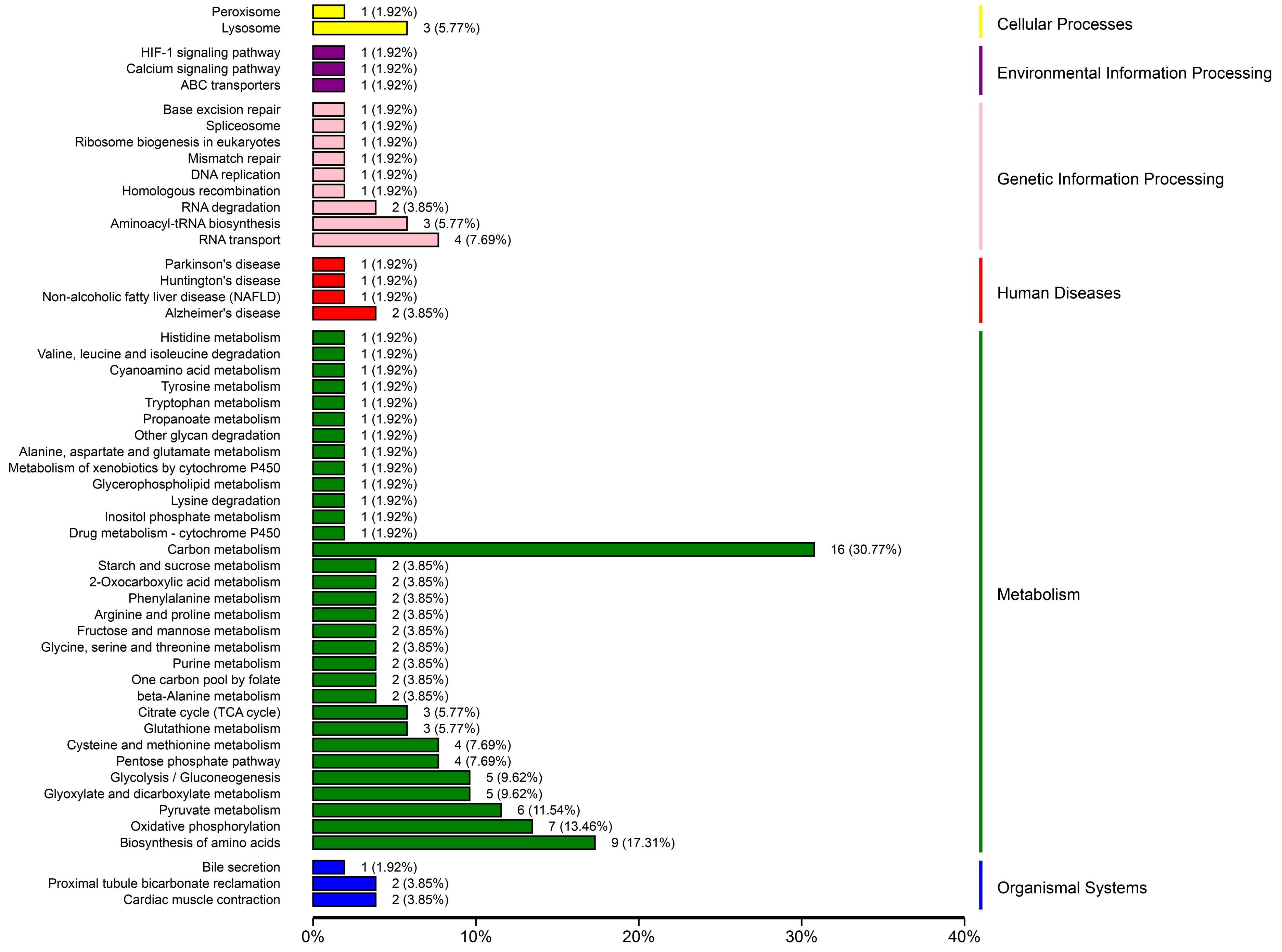

Fig. S11 KEGG pathway analysis of DEGs of *S. graminum* fed on WDV-infected wheat.

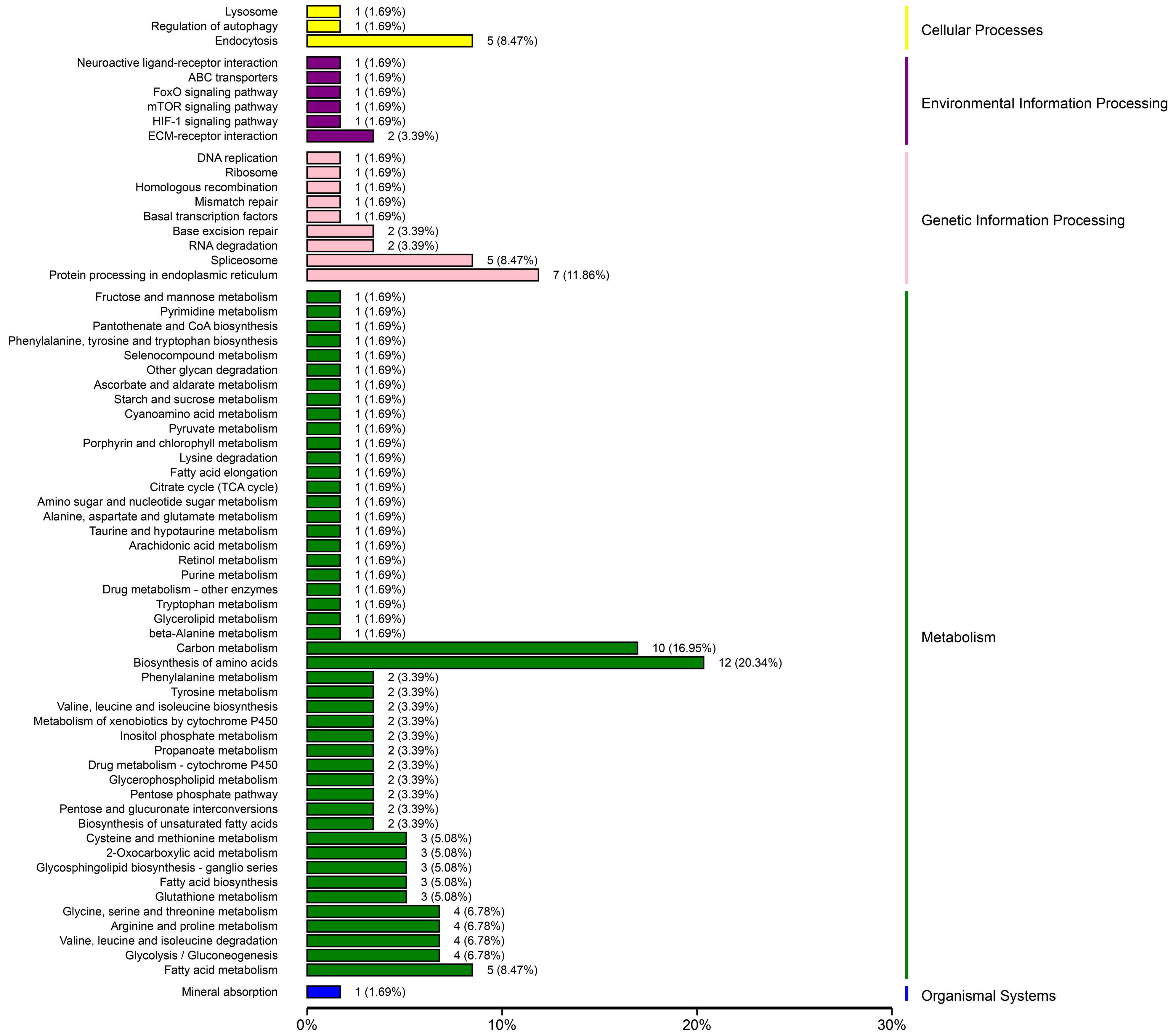

Fig. S12 KEGG pathway analysis of DEGs of *R. padi* fed on BYDV-infected wheat.

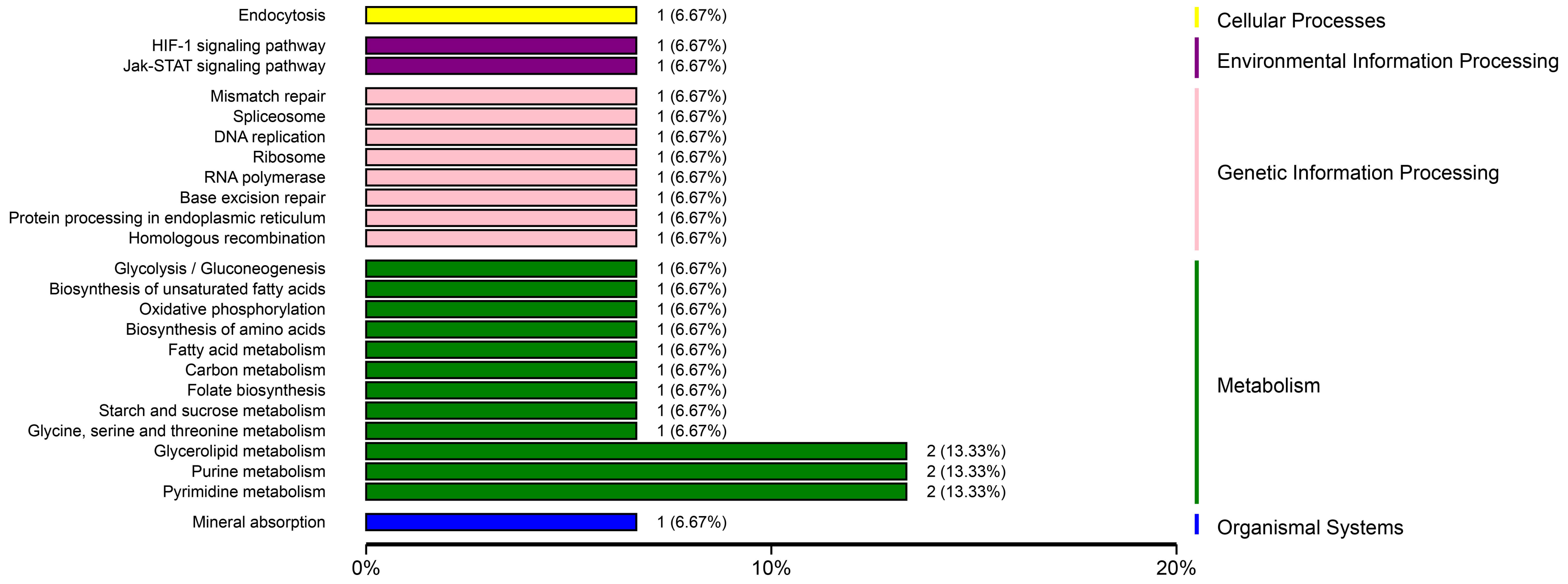

Fig. S13 KEGG pathway analysis of DEGs of *R. padi* fed on WDV-infected wheat.

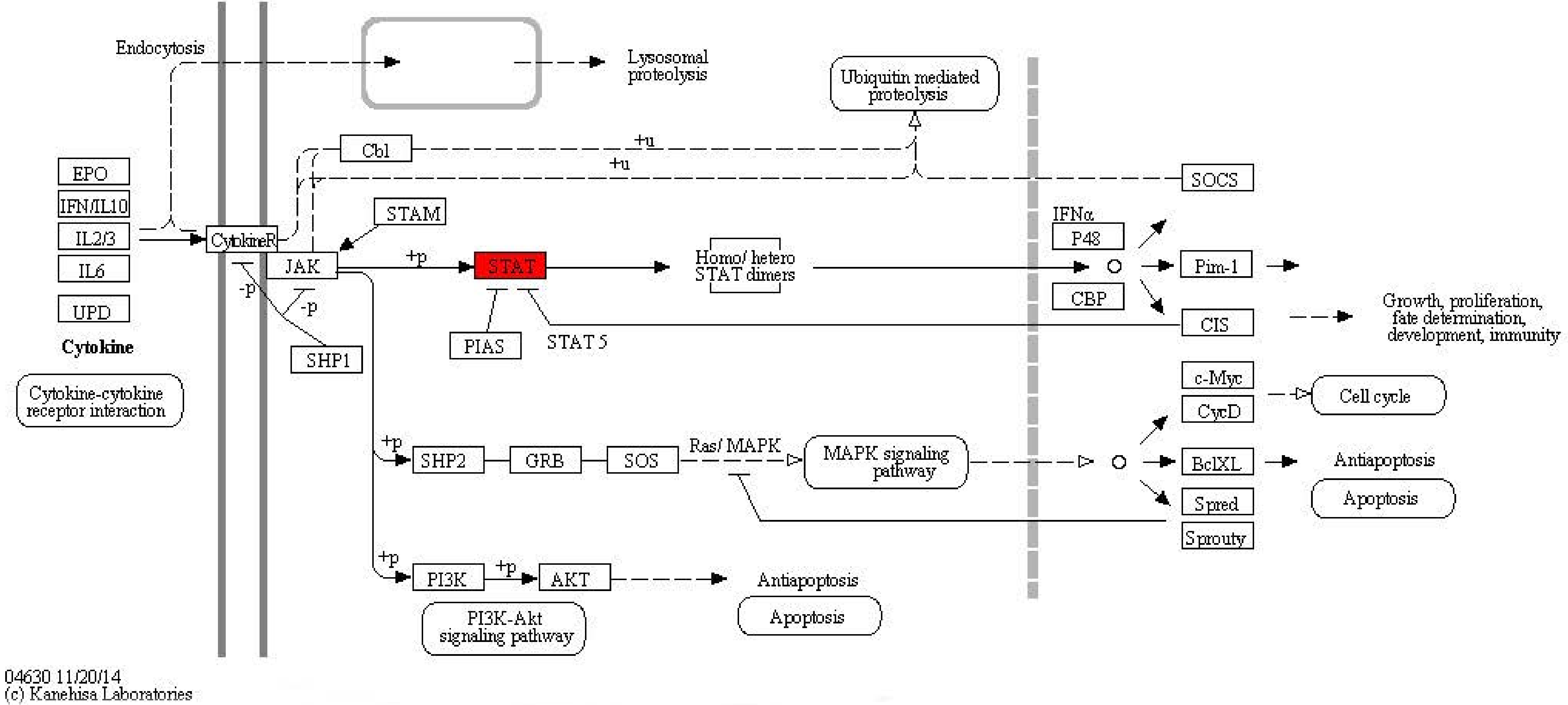

Fig. S14 JAK/STAT signaling pathway
